# Cortical activity during painful and non-painful stimulation over four lower limb body sites: a functional near-infrared spectroscopy study

**DOI:** 10.1038/s41598-025-87699-w

**Published:** 2025-02-11

**Authors:** Jiawen Liao, Stefano Silvoni, Simon Desch, Angela Serian, Jamila Andoh, Herta Flor

**Affiliations:** 1 Department of Neuropsychology and Psychological Resilience Research, Central Institute of Mental Health, Medical Faculty Mannheim, Heidelberg University, J 5, 68159 Mannheim, Germany; 2 Clinical Psychology, Department of Experimental Psychology, Heinrich Heine University Düsseldorf, Düsseldorf, Germany; 3 Department of Psychiatry and Psychotherapy, Central Institute of Mental Health, Medical Faculty Mannheim, Heidelberg University, Mannheim, Germany

**Keywords:** Pain, fNIRS, Groin, Knee, Electrical stimulation, Neuroscience, Psychology

## Abstract

Functional near-infrared spectroscopy (fNIRS) holds potential utility as a measure of neural correlates of pain. However, most studies have focused on upper limb stimulation, with limited investigation into lower limbs. In this study, we utilized fNIRS to observe brain changes in oxyhemoglobin levels during painful and non-painful electrical stimulation of various lower limb sites (bilateral groins and knees) in 16 healthy participants. Additionally, we explored perceptual responses to painful and non-painful electrical stimulation across these lower limb sites. Our findings showed no significant main effect of stimulation across different body sites on oxyhemoglobin activity. However, the interaction between body sites, stimulation modalities, and brain regions significantly influenced oxyhemoglobin activity. Specifically, we found a decrease of neuro-metabolic activity in prefrontal and bilateral primary somatosensory cortices during painful stimulation of the left groin compared to non-painful stimulation, whereas an increase of neuro-metabolic activity was observed during painful stimulation of the right knee. Our findings also revealed that stimulation intensity was notably lower for bilateral knees compared to the left groin. These findings underscore the potential and feasibility of utilizing fNIRS to investigate pain mechanisms related to stimulation across distinct lower limb regions.

**Supplementary Information:**

The online version contains supplementary material available at 10.1038/s41598-025-87699-w.

## Introduction

The occurrence of pain is a complex and multifaceted psychophysiological process involving multiple psychobiological components. Acute pain can be induced by various stimuli^1^ and neuroimaging can aid in unraveling the central nervous system factors related to pain perception. Prior neuroimaging studies have demonstrated that painful stimuli elicit responses across a wide array of cortical and subcortical networks^2^. For instance, Freund et al.^3^ observed that when healthy participants were exposed to painful thermal stimulation to the left or right side of the hand, functional magnetic resonance imaging (fMRI) consistently showed activation in the right anterior insula, the ipsilateral sensorimotor cortex, and the bilateral posterior insula. In another fMRI investigation^4^, the bilateral secondary somatosensory cortex exhibited notable activation when both painful and non-painful electrical stimulation were administered to healthy participants. Furthermore, other published brain imaging studies^5–7^ have also provided evidence that structures such as the prefrontal cortex (PFC), dorsolateral PFC (DLPFC), and cingulate cortex are linked to the modulation and perception of pain. Based on the current scientific literature^8^, the brain regions implicated in pain processing encompass the primary (S1) and secondary (S2) somatosensory cortex, the primary motor cortex (M1), DLPFC, the cingulate area, the insula, as well as subcortical structures such as the thalamus and hypothalamus. Typically, the cingulate cortex, insula, and limbic system areas are implicated in the emotional aspects of pain^9^. S1 and S2 are typically associated with the sensory dimension of pain^10^, while cognitive aspects of pain are linked to the frontal and parietal regions of the brain^11^.

FMRI studies have enabled the precise monitoring and comprehension of pain-related brain regions and pain-related alterations in brain function and metabolism^12^. However, fMRI can be costly, cannot be carried out at bedside, has contra-indications for persons with metal implants, and necessitates participants to remain relatively motionless during data acquisition, which can pose challenges for experimental procedures and recruitment^13^. Consequently, functional near-infrared spectroscopy (fNIRS) has become a valuable alternative over the past two decades.

FNIRS is a non-invasive brain imaging technique with few restrictions on its use and has the advantage of portability (the equipment can be brought to the patient)^14^. It can be used to determine hemodynamic and metabolic changes associated with brain activity by measuring changes in oxygenated hemoglobin (HbO2) and deoxyhemoglobin (HbR) concentrations in real-time^15,16^. However, the depth detection capability of fNIRS is typically influenced by specific instrument settings and measurement conditions. In general, fNIRS predominantly measures activity in the cerebral cortex, with a depth range of a few centimeters. Its capacity to measure deep brain structures is limited compared to fMRI^17^. Therefore, it is notable that existing studies on fNIRS and pain primarily focus on the PFC and S1. For instance, Becerra et al.^18^ found that PFC and S1 activation was observed bilaterally when painful thermal stimulation was given on the dorsum of the right hand. Another fNIRS study reported PFC activation in response to a mechanical painful stimulus applied to the right index finger^19^. However, contrasting results emerged in a different study where a painful mechanical stimulus was applied to the gingiva^20^. Additionally, some studies utilizing electrical stimulation found that PFC exhibited deactivation when pain was induced in the thumbs of healthy participants^21,22^. Using a 2-channel NIRS setup, one study found that when applying electrical stimulation to the right ventral arm, there were no significant differences in changes in blood oxygen concentration within PFC, regardless of whether the stimulation was painful or non-painful^23^. Hence, it is evident that the present research findings exhibit some inconsistencies. These variations may be attributed to differences in the intensity or nature of the applied stimuli, or they could be linked to variations in the different body sites. Additionally, it is noteworthy that existing research predominantly focuses on measuring upper limb areas such as hands, arms, and thumbs. There is a dearth of studies examining lower limb regions.

Lower limb pain is usually localized at the joints, particularly in regions such as the groin and knee^24^. The primary causes include knee osteoarthritis, inguinal hernia, phantom limb pain, and sport or exercise-induced musculoskeletal injuries^25–28^. While there are several fNIRS studies investigating pain in patients with knee osteoarthritis^29,30^, research focusing on groin pain is scarce. Additionally, as mentioned earlier, the inconsistent findings in existing fNIRS studies on pain may stem from differences in the body sites chosen for stimulation. Investigating multiple body sites can help clarifying whether cortical responses to pain vary depending on the anatomical location of the stimulus. This is particularly relevant in amputees, as alterations in sensory perception were detected at sites remote from the amputation, and these changes showed a notable correlation with the experience of phantom pain^31^. Therefore, we designed this pilot study as a foundation for future investigations into lower limb amputees experiencing phantom limb pain. Yennu et al.^32^ utilized fNIRS to investigate the activation of the cerebral cortex when three different body sites over the right forearm, right temporomandibular joint, and left forearm were stimulated by noxious thermal pain. They found that the hemodynamic activity in the PFC exhibited consistent temporal and spatial patterns in response to acute thermal stimuli across all three body sites. However, their study was confined to the upper body region, highlighting the need for broader investigation across different body areas.

The primary objective of our study was to employ fNIRS to monitor changes in blood oxygen concentration within the PFC and S1 when healthy persons were subjected to varying intensities of electrical stimulation (both painful and non-painful). Based on a comprehensive review^33^ of previous research findings regarding the stimulation of other body sites under external sensory stimulation, the discriminative effect of S1 on sensations, as well as the inhibition of amygdala activity and default mode network deactivation, are likely contributors to reduced blood flow in the PFC area. Therefore, our hypothesis was that stimulation of the lower limb sites would evoke increased neuro-metabolic activity in S1 in response to painful stimuli compared to non-painful stimuli. Additionally, we anticipated decreased neuro-metabolic activity in prefrontal regions during painful stimuli compared to non-painful stimuli. We also aimed to investigate these changes across four distinct lower limb regions: both groins and both knees. However, based on the findings from previous studies mentioned earlier^32^, we hypothesized that there would be no significant differences in activation patterns across these different body sites.

## Methods

### Participants

Sixteen right-handed healthy persons (8 male) between 21 and 30 years of age (mean = 24, SD = 3.4) participated in the study. They were recruited via flyers posted on the official website of the Central Institute of Mental Health and distributed throughout the university campus. The ethics committee of the Medical Faculty Mannheim, Heidelberg University, Germany, approved the study (ethics approval number: 2014–596 N-MA). All methods were performed in accordance with the relevant guidelines and regulations. Each participant gave written informed consent before the experiments. Exclusion criteria were: prior history of diseases of vital organs, brain tumors or other cerebral disorders or trauma, and history of mental illness.

### Experimental design

The study design consisted of the following procedure: (a) assessment of perception and pain thresholds as well as pain tolerance to electrical skin surface stimulation of the left/right groin and left/right knee; (b) determination of painful and non-painful stimulation intensities; (c) delivery of painful and non-painful stimulation using a block-design experimental stimulation modality with pre- and post-assessment of perceived stimulation intensity through a rating using a visual analogue scale^34^ (VAS). Throughout the experiment, fNIRS was utilized to monitor changes in blood oxygen concentration.

**Fig. 1:**
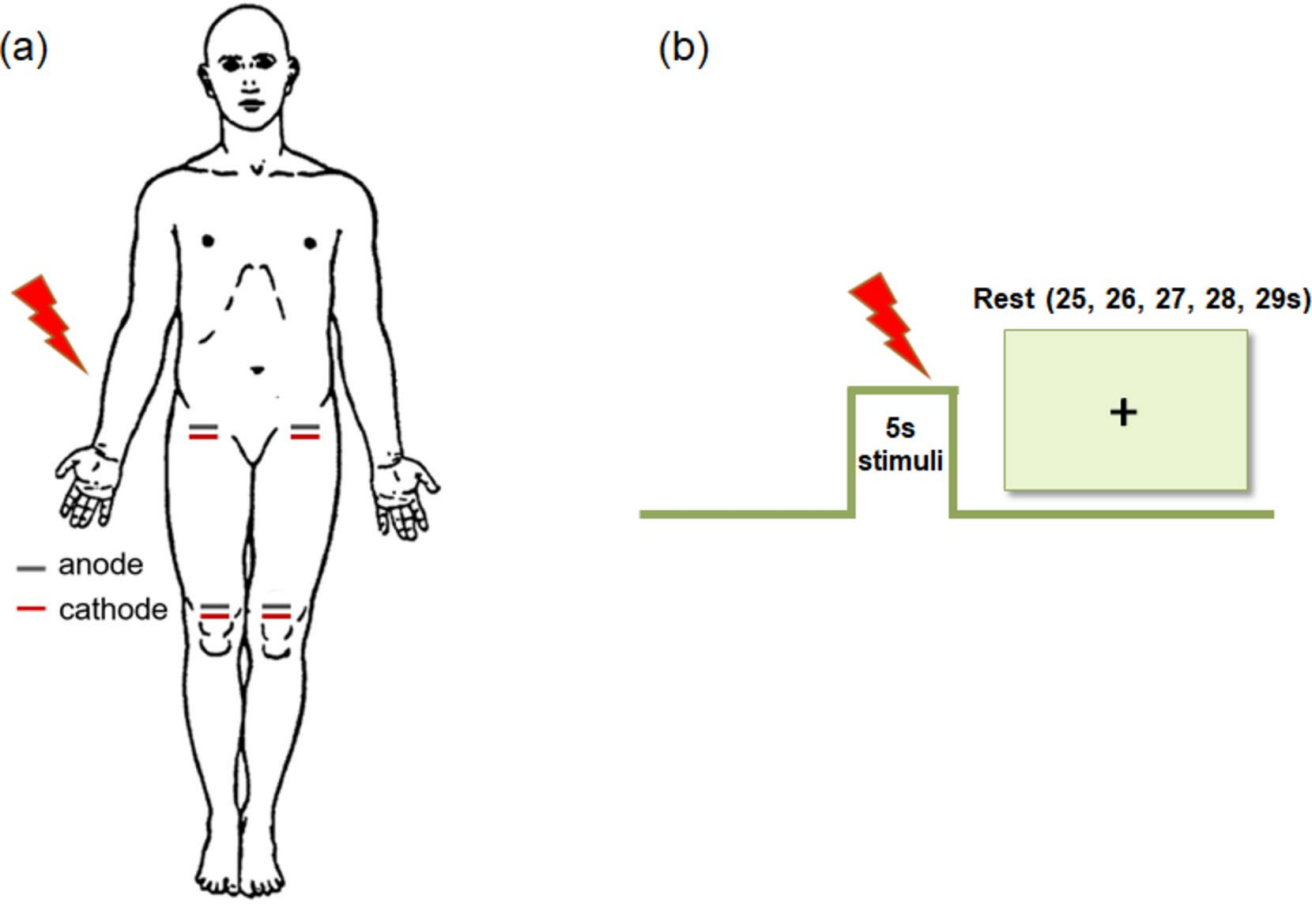
(**a**) Schematic illustration of the stimulation at the various body sites. The electrical stimulation was applied at left and right groins and both knees via an anode and cathode at a distance of about one centimeter between them. The blue line represents the anode. The red line represents the cathode. And the lightning bolt represents the electrical stimulation. (**b**) Representation of one stimulation block and a rest block. Each stimulation modality consisted of 6 stimulation blocks of 5 s, each separated by 6 rest blocks of pseudo-randomised duration (25 to 29 s). The lightning bolt represents the electrical stimulation.

For somatosensory stimulation, we applied electrical stimuli, using a copper electrode connected to a Digitimer stimulator (DS7A, Digitimer, Hertfordshire, England) with monophasic square-wave pulses of 0.2 ms at a 5 Hz frequency. Before the experimental stimulation modality, we tested the participants’ perception and pain thresholds as well as pain tolerance to find the optimal painful and non-painful stimulation intensities. Similar to previous studies^35,36^, for each threshold, we calculated the average value of the stimulation intensity (mA) of three ascending series. The participants were instructed to press a button when they started to feel the stimulus (perception threshold), when the stimuli started to hurt (pain threshold), and when they could no longer tolerate the stimuli (pain tolerance). We preset the non-painful stimulation intensity at 50% between perception and pain thresholds, and the painful stimulation intensity was preset at 50% between pain threshold and pain tolerance. Next, to ensure the applicability of the obtained stimulus intensities in the experimental stimulation modalities, participants evaluated the provided stimuli, and the stimulation intensities were further calibrated. The painful and non-painful stimulus intensities were adjusted to a perceived stimulation intensity around 75% and 25% of a VAS scale, respectively. The VAS scale was presented as a 600-pixel (16.2 cm) vertical line, with “No sensation” at the bottom, “Pain sensation” in the middle, and “Extreme pain” at the top. Participants were instructed to evaluate the perceived electrical stimulation by marking a point on this line to reflect their experience, which could later be converted into numerical values of 0 to 100. We defined the values between “No sensation” and “Pain sensation” as non-painful stimulation intensity, and the values between “Pain sensation” and “Extreme pain” as painful stimulation intensity. We assessed the response to stimulation in four body sites: left and right groins, and left and right knees. The stimulation site on the groin is at the junction between the trunk and the thigh, while on the knee, it is above the patella, as shown in Fig. 1(a). According to a previous study^21^ and the aim of our study, a block-design experimental stimulation modality was employed to deliver painful and non-painful stimulation to the participants. Each body site was tested using two stimulation modalities: a painful stimulation and a non-painful stimulation. Each stimulation modality consisted of six 5-second stimulation blocks, interspersed with six rest blocks of pseudo-randomized durations (ranging from 25 to 29 s). This pseudo-randomization was designed to prevent participants’ expectations and mitigate the effects of pain anticipation in the recorded signals, as shown in Fig. 1 (b). Before and after the stimulation modality, the participants rated the perceived stimulation intensity by a VAS rating. The order of stimulation modalities was counterbalanced across the participants. Before the first stimulation block, we recorded 25 s of baseline neuro-metabolic activity during which the participant was asked to relax and look at a cross displayed on a screen. During the experiment, the participants kept their eyes open, concentrating on the stimulation, while looking at the screen where instructions were presented.

### FNIRS data recording

**Fig. 2:**
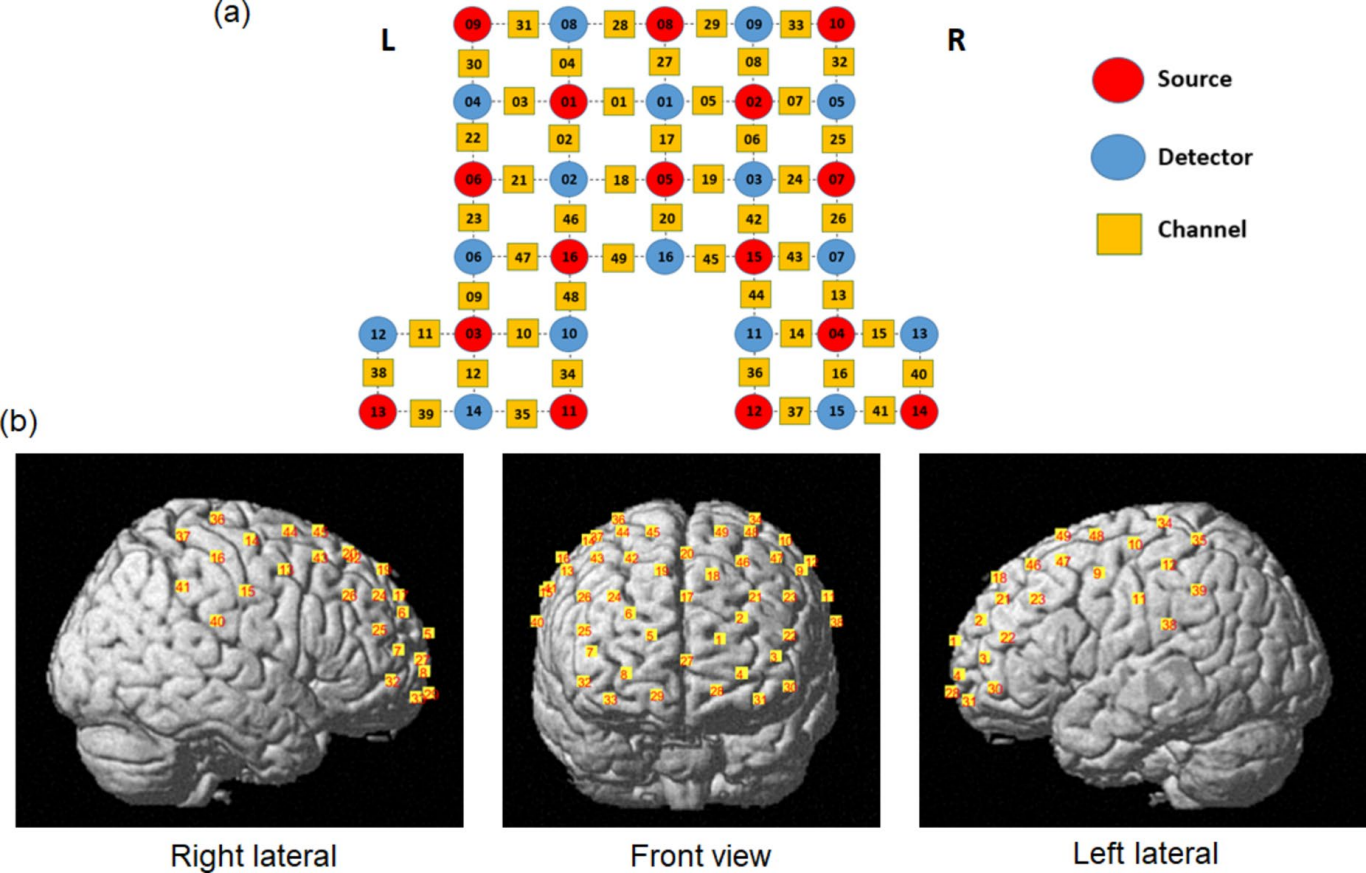
(**a**) fNIRS optode and channel placement over the bilateral primary somatosensory cortex and prefrontal cortex within the international 10–10 standard system. The red and blue dots represent the location of light sources and detectors, respectively. The yellow squares represent the location of the channels. Sources 03, 04, and 05 represent C3, C4, and Fz, respectively. (**b**) The co-registration of the 49 channels on a standard human brain template.

Cerebral hemodynamic activity was recorded using a non-invasive multichannel functional near-infrared spectrometer operating at 760 and 850 nm wavelengths (NIRScout 24*24, NIRx Medical Technologies, Berlin, Germany). NIRStar software was used for data recording (NIRx Medizintechnik GmbH, Berlin, Germany). The signal sampling rate was 3.91 Hz. We employed a MATLAB-based toolbox^37^ (fOLD—fNIRS optode location decider) to identify the functional brain region of interest for optode-based neuro-metabolic recording. This software accurately estimates spatial brain mapping of the standard electroencephalography (EEG) 10–10 systems on associated Brodmann areas^38^. According to this method, we identified the Brodmann areas 1, 2, 3, 9, 10, 11, and 46 as the regions of interest in this study. In fOLD software, specificity is defined as the proportion of photon fluence within the regions of interest relative to the total fluence across all regions covered by the source-detector pair. This metric represents the degree to which the photon distribution is concentrated in the regions of interest, providing anatomical specificity for channels corresponding to these regions. In this study, a specificity threshold of 10% was applied to select channels that received at least 10% of the total fluence from the regions of interest. The channels were further adjusted and visualized using NIRSite software (NIRx Medical Technologies, Glen Head, NY). The probe consisted of sixteen light sources and sixteen detectors. Optodes were placed on the prefrontal and bilateral primary somatosensory areas, forming a 49-channel setup with an inter-probe distance of a maximum of 3 cm. The placement of optodes and channels were shown in Fig. 2. Detailed information for each channel can be found in Table S10 of supplementary materials. The infrared light sources and detectors were placed in an EEG cap (EASYCAP GmbH, Germany). The cap size was determined depending on the head circumference of participants (54 cm, 56 cm, 58 cm, and 60 cm).

### Data processing, analysis and statistics

To ensure the quality of our data, we first examined the results of the VAS rating used in our experiment. We then eliminated any fNIRS data where participants reported feeling pain in response to non-painful stimuli and where non-painful stimuli were reported as painful. After this screening process, the sample size of analyzed data was 13 participants for the left groin, 15 for the right knee, 16 for the right groin, and 16 for the left knee. These data were then processed and analyzed separately for the painful and non-painful stimulation. We computed the coefficient of variation (CV) for each channel by dividing the standard deviation of the channel data by the mean and multiplying by 100. The CV threshold was set at 15% to minimize the impact of physical artifacts and ensure the quality of the fNIRS data^39^. No channels were excluded during this process. Subsequently, to explore the spatial correlations under different stimulation madalities, the NIRS data were analyzed using the open-source software NIRS-SPM^40^, implemented in Matlab R2020a. The raw intensity data recorded by NIRStar software were first converted to optical density values using NIRS-SPM’s built-in conversion function. We preprocessed the data using the wavelet-minimum description length (wavelet-MDL) and the low-pass filter of NIRS-SPM. The wavelet-MDL method, using a wavelet filter with a support size of 9, effectively removed various types of noise and artefacts, including cardiac activity, respiration, and vaso-motion, thereby providing a more accurate estimation of the brain signal^41^. In NIRS-SPM, the low-pass filter was applied by selecting the ‘hrf’ (hemodynamic response function) option, with a 6 dB cut-off frequency of 0.1 Hz to filter out high-frequency noise. We employed a pre-coloring method to estimate the temporal correlation of fNIRS data^42^. Subsequently, we utilized a general linear model to calculate parameter estimates of the neuro-metabolic activity through convolution with the canonical hemodynamic response function (with time and dispersion derivatives) for each of the two experimental stimulation modalities separately (painful and non-painful stimulation) referred to the neuro-metabolic activity of all intervals without stimulation. We obtained the beta (β) value of each channel representing the magnitude of the task response. Then, according to the regions of interest in this study, we grouped channels into different brain regions to obtain the average β value of each brain region. Channel 9, 10, 11, 12, 34, 35, 38 represent the left S1 cortex. Channel 13, 14, 15, 16, 36, 37, 40 represent the right S1 cortex. And channel 1, 2, 3, 4, 5, 6, 7, 8, 17, 18, 19, 20, 21, 22, 23, 24, 25, 26, 27, 28, 29, 30, 31, 32, 33, 42, 43, 46 and 47 represent the PFC area. The β value calculated by this method represents an increase (activation) or a decrease (deactivation) of the regional neuro-metabolic activity during stimulation with respect to the non-stimulation intervals. On a linear scale, positive β values indicate activation, while negative β values denote deactivation.

Data analysis was conducted using Matlab R2020a. Somatosensory intensities were analyzed using a two-way analysis of variance (ANOVA), while the VAS ratings and β values were examined with repeated-measures ANOVA. For the β values analysis, due to the unbalanced sample size, we handled missing data using three approaches: deleting subjects with missing data, replacing the missing data with the mean, and replacing it with the median. The results were consistent across all three methods. Therefore, the findings of this analysis are primarily based on available data with complete set of β values for each of the four body sites. The effect size for the ANOVA was calculated using partial eta-squared (*η*^2^), and the magnitude of the effect was interpreted based on the criteria: small (*η*^2^ = 0.01), medium (*η*^2^ = 0.06), and large (*η*^2^ = 0.14)^43^. The post-hoc tests for stimulation intensities and VAS rating were controlled for multiple comparisons using the Tukey-Kramer test. The post-hoc tests for the β values were controlled using the false discovery rate (FDR). Cohen’s *d* was used to calculate the effect size for each contrast. Effect sizes were interpreted as small (*d* = 0.2), medium (*d* = 0.5), and large (*d* = 0.8)^43^. The normality test was performed using Shapiro-Wilk normality test, see Supplementary Tables S1, S3 and S5. Although the normality assumption was not confirmed, a repeated measures ANOVA was used for modeling the data, due to its robustness against violations of normality^44^. We also tested data transformation to normalize the data but were not successful. Where appropriate, we used the non-parametric equivalent to ANOVA contrasts. There were no deviations from the parametric versions. BrainNet Viewer^45^, an open-source toolbox, was used for data visualization. The significance level was set at 0.05. The results section highlights HbO2 concentration changes, which are generally more sensitive to task-related neural activity compared to HbR changes^46–48^.We however also include findings related to HbR, which are now provided in the supplementary materials (see Supplementary Fig. S1 and Table S9).

## Results

### Stimulation intensity

**Fig. 3:**
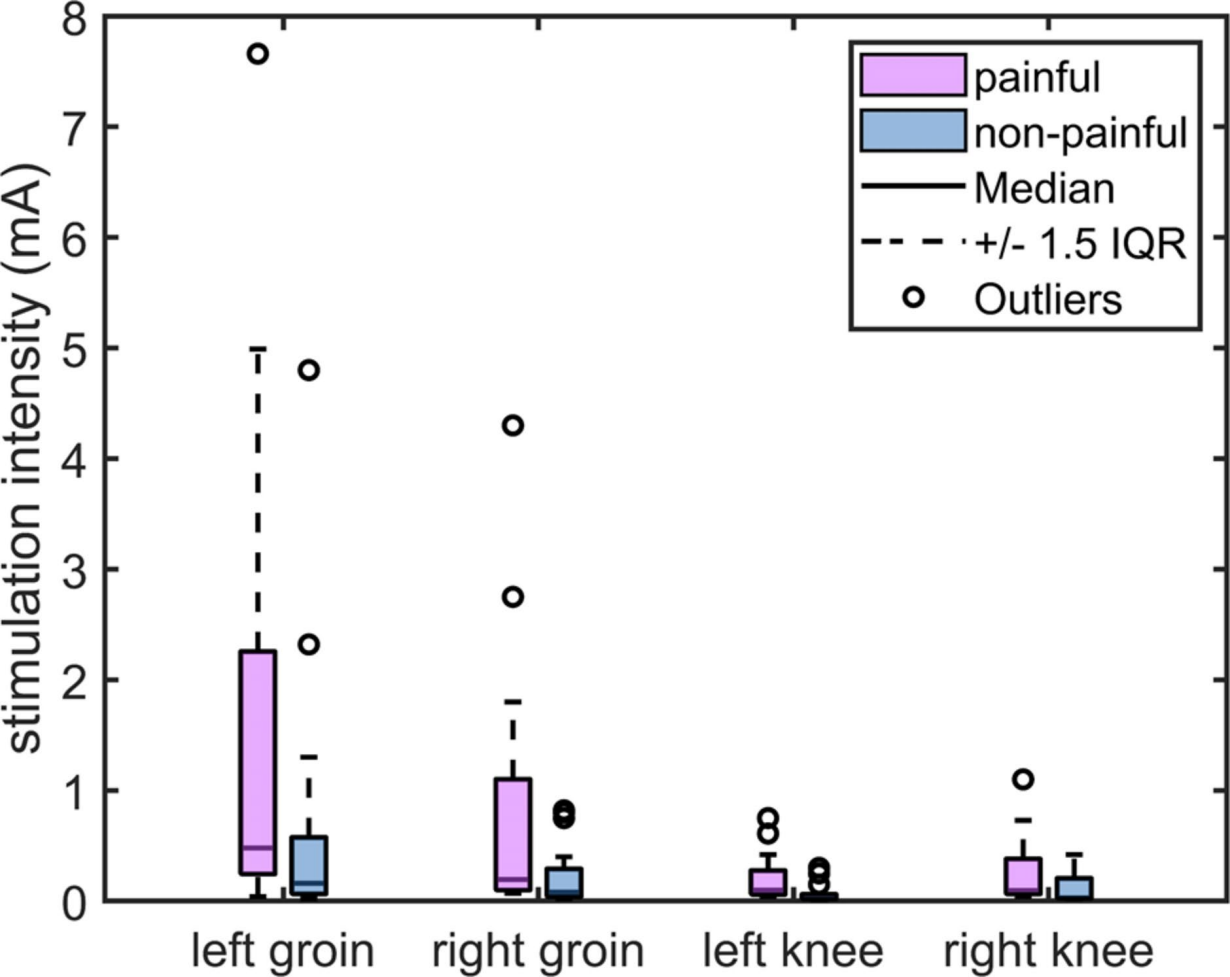
Stimulation intensity (mA) of each body site under the two stimulation modalities. Boxplots are used to display the painful stimulus values (represented by pink boxes) and non-painful stimulus values (represented by blue boxes) at each of the four locations. The central line within each box represents the median. The edges of the box correspond to the 25th and 75th percentiles, defining the interquartile range (IQR). Whiskers extend to the most extreme data points within 1.5 times the IQR from the first and third quartiles. Outliers are depicted as circles outside the boxes.

Descriptive statistics of stimulus intensities over the two stimulation modalities and the four different body sites are presented in Fig. 3 and Table S1. The two-way ANOVA with stimulation intensities as a dependent variable, based on a total of 16 observations, revealed main effects of body sites and stimulation modality (painful vs. non-painful) on the stimulation intensities (body sites: *F*_*3*_ = 6.6, *p* < 0.001, *η*^2^ = 0.142; stimulation modality: *F*_*1*_ = 6.2, *p* = 0.014, *η*^2^ = 0.049), but no significant interaction between these two factors (*F*_*3*_ = 1.2, *p* = 0.330, *η*^2^ = 0.028). Post-hoc Tukey test revealed that the stimulation intensities at both knees were significantly smaller than those assessed at the left groin (left groin vs. left knee: *p* < 0.001; left groin vs. right knee: *p* < 0.05), see Supplementary Table S2for details.

### VAS rating

**Fig. 4:**
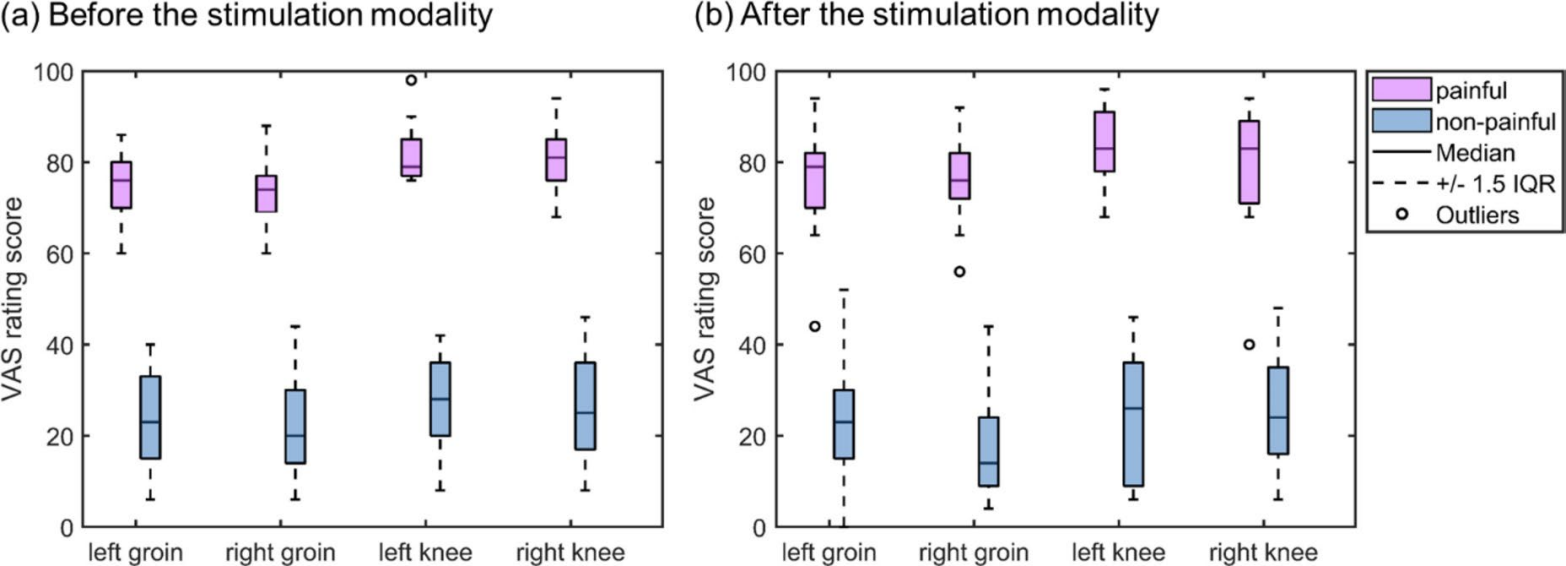
The visual analogue scale (VAS) ratings of each body site under the two stimulation modalities. Boxplots are used to display the VAS ratings under the painful stimulation (represented by pink boxes) and non-painful stimulation (represented by blue boxes) at each of the four locations. The central line within each box represents the median. The edges of the box correspond to the 25th and 75th percentiles, defining the interquartile range (IQR). Whiskers extend to the most extreme data points within 1.5 times the IQR from the first and third quartiles. Outliers are depicted as circles outside the boxes. The (**a**,** b**) show the different rating times (**a** before the stimulation modality; **b** after the stimulation modality).

**Fig. 5:**
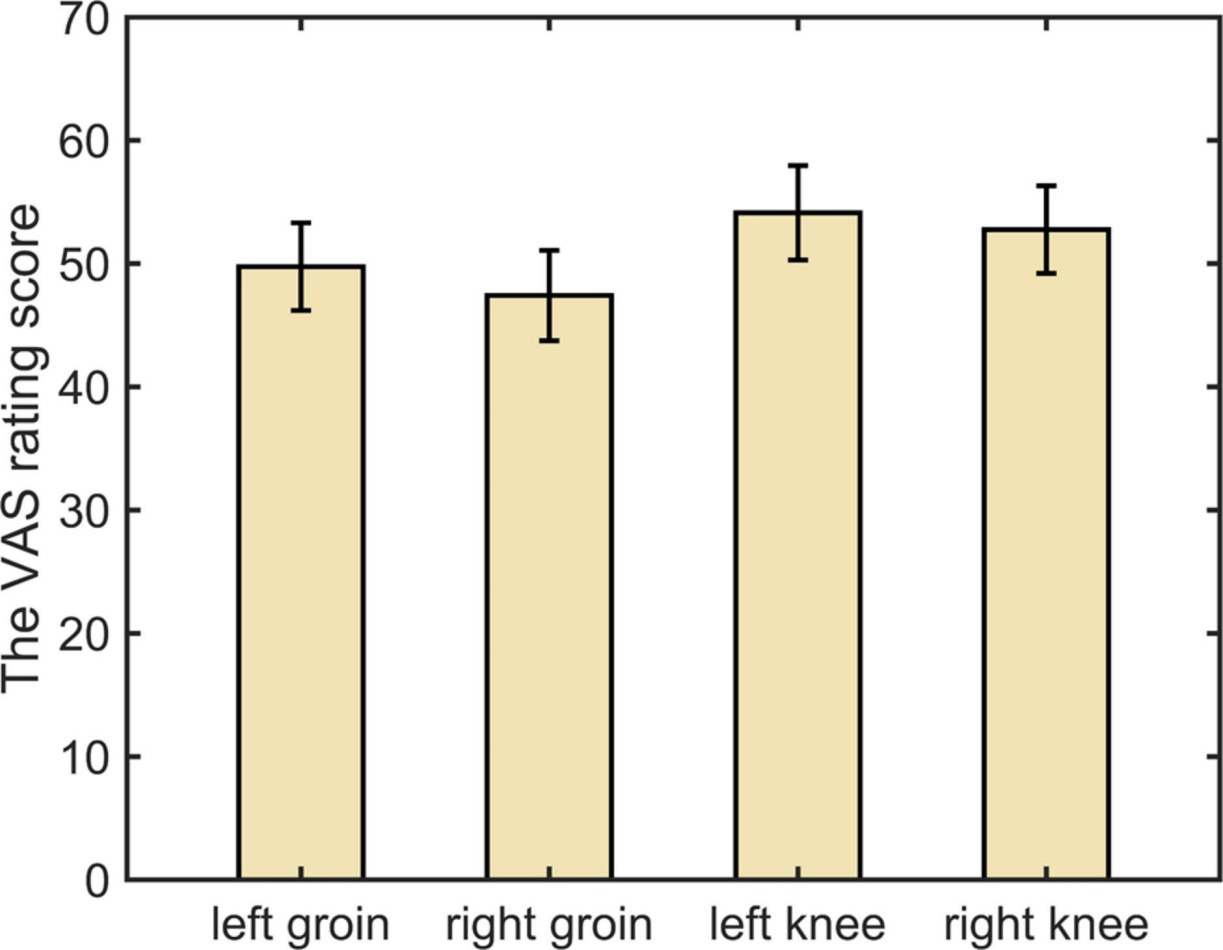
Bar plots showing the mean visual analogue scale (VAS) rating across body sites. Error bars represent the standard error of the mean (SE).

Descriptive statistics of the VAS ratings over the two stimulation modalities and the four body sites are represented in Fig. 4 and Table S3. The repeated-measures ANOVA of the VAS rating, with a total of 16 observations, showed a main effect of body sites and a main effect of stimulation modality (painful vs. non-painful), but no significant main effect of time (before the stimulation modality vs. after the stimulation modality) (body sites: *F*_*3*_ = 3.6, *p* = 0.022, *η*^2^ = 0.191; stimulation modality: *F*_*1*_ = 413.4, *p* < 0.001, *η*^2^ = 0.965; time: *F*_*1*_ = 0.1, *p* = 0.720, *η*^2^= 0.009) (see Table 1). The post-hoc Tukey test depicted in Fig. 5 revealed no significant differences after multiple comparisons correction among the body sites. However, the VAS rating for the right groin was lower than that for the left knee, showing a trend, as indicated by a *p*-value of 0.05; see Supplementary Table S4 for details.

**Table 1:**
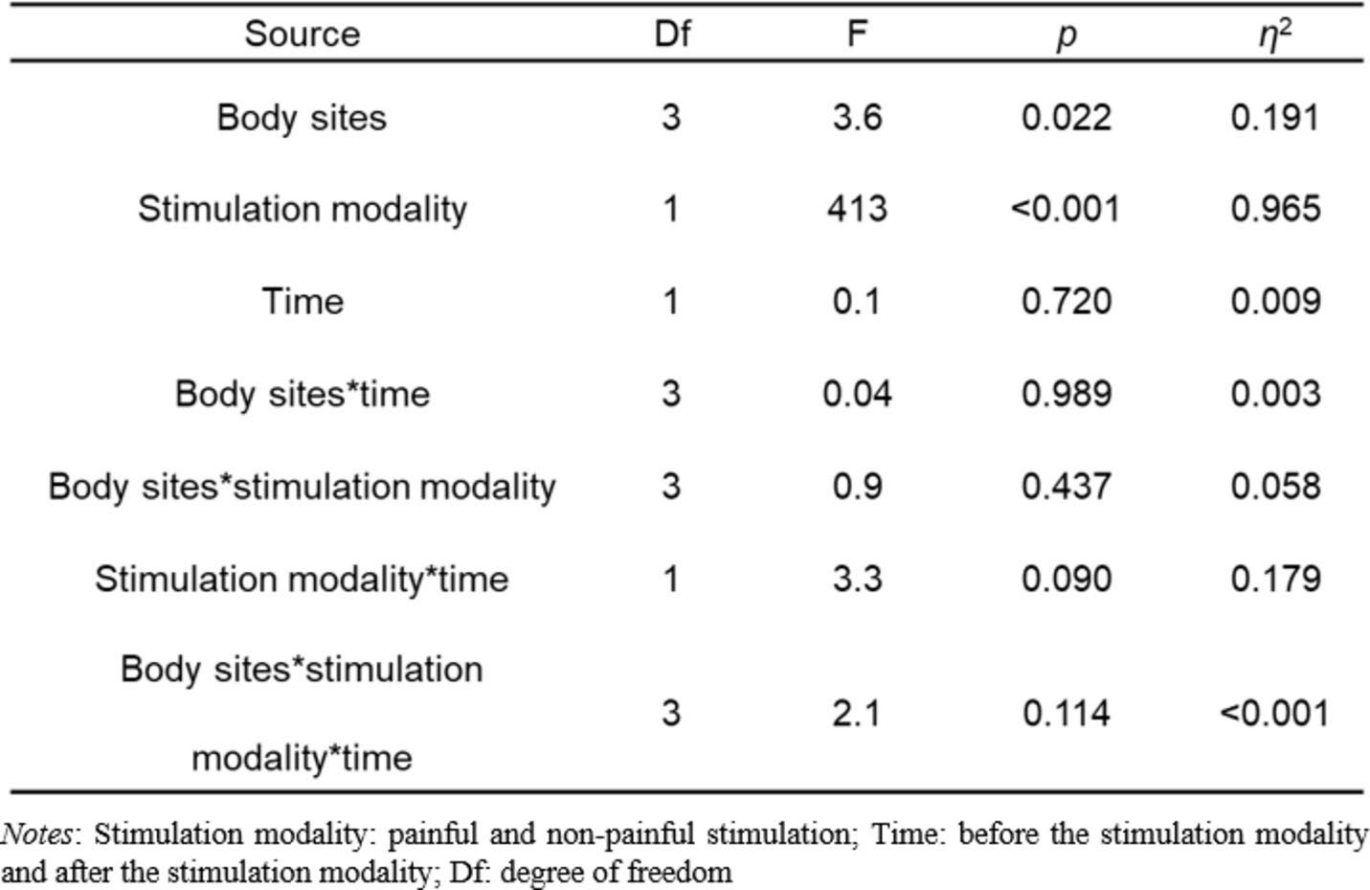
Repeated-measures ANOVA results for visual analogue scale (VAS) rating.

### Analysis of brain activation

We utilized the general linear model to generate interpolated topographical maps for the different body sites exposed to painful and non-painful stimuli. This enabled us to examine how various brain regions were activated or deactivated in response to these stimuli. As described in the method section, a positive β value reflects the activation of regional neuro-metabolic activity during stimulation compared to non-stimulation intervals, while a negative β value indicates its deactivation. The repeated-measures ANOVA of the β values, with a total of 12 observations, showed a main effect of brain region (*F*_*2*_ = 9.2, *p* = 0.001, *η*^2^ = 0.455), but no significant main effect of body sites and stimulation modality (painful and non-painful) (Body sites: *F*_*3*_ = 0.2, *p* = 0.921, *η*^2^ = 0.014; Stimulation modality: *F*_*2*_ = 0.10, *p* = 0.761, *η*^2^ = 0.009). The interaction between body sites, stimulation modality, and brain region was significant (*F*_*6*_ = 3.1, *p* = 0.009, *η*^2^ = 0.221), indicating that the combined effects of these factors significantly influenced the β values. See the Supplementary Table S6 for the detailed information. The results of the post-hoc multiple comparisons between the β values for each brain region can be found in Supplementary Table S7.

**Fig. 6:**
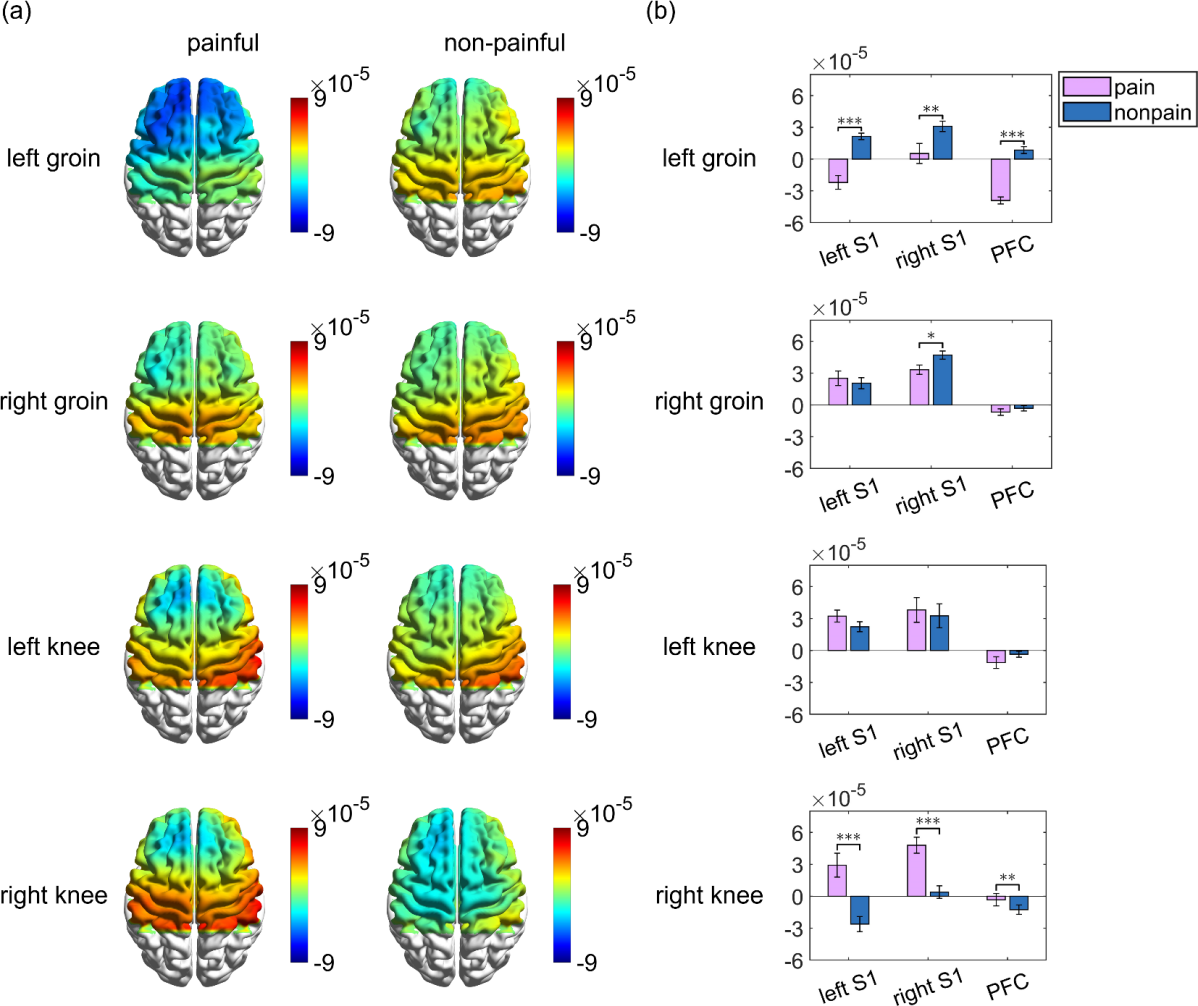
(**a**) Topographic images of group-averaged cortical deactivations and/or activations during painful (left) and non-painful (right) stimulation modalities given on each body site (left groin, right groin, left knee and right knee). (**b**) Comparisons of the group-averaged β values of different brain regions when stimulating different body sites. The pink bar represents the painful stimuli, and the blue bar represents the non-painful stimuli. Left S1 means left primary somatosensory area, right S1 means right primary somatosensory area, and PFC means prefrontal area. Error bars represent the standard error of the mean. (*p < 0.05, **p < 0.01 and ***p < 0.001).

Figure 6 showed the results of post-hoc multiple comparisons of the β value of each brain region under different stimulation modalities (painful and non-painful) for each body site. We observed the activation of bilateral S1 and PFC areas in response to a non-painful stimulation on the left groin. During painful stimulation of the left groin, we noted deactivation in the ipsilateral S1 cortex and PFC. Figure 6 (b) clearly indicated a significant increase in oxyhemoglobin levels in bilateral S1 cortex and PFC during non-painful stimulation compared to painful stimulation of left groin. When we applied electrical stimulation to the right groin, we observed activation in the bilateral S1 cortex and deactivation in the PFC, independent of the stimulation modality. However, the ipsilateral S1 cortex exhibited greater activation in response to non-painful stimuli compared to painful stimuli, as shown in Fig. 6 (b). When we stimulated the left knee, we observed activation in the bilateral S1 cortex and deactivation in the PFC. There is no statistical significance observed in the alterations within these three distinct brain regions when comparing activity patterns of the cerebral cortex under painful and non-painful stimulation, as depicted in Fig. 6 (b). As can be seen in Fig. 6 (a), we saw activation in the bilateral S1 cortex and deactivation in the PFC when the painful stimulation were provided on the right knee. We observed activation in the ipsilateral S1 cortex and deactivation in the contralateral S1 cortex, the PFC, during non-painful stimulation of right knee. We found a significant increase in oxyhemoglobin levels in these three brain regions during the painful stimulation when compared to non-painful stimulation; see Fig. 6 (b). See the Supplementary Table S8 for the statistical results of Fig. 6 (b).

## Discussion

This study examined changes in oxyhemoglobin levels in the cerebral cortex when healthy persons were subjected to painful and non-painful stimuli administered at four lower extremity sites using fNIRS. We found that there was no significant main effect of body sites and stimulation modalities on oxyhemoglobin activity. However, brain regions and the interaction between body sites, stimulation modalities, and brain regions significantly influenced oxyhemoglobin activity. Specifically, painful stimulation of the left groin resulted in deactivation of the PFC and the bilateral S1 compared to non-painful stimulation. In contrast, painful stimulation of the right knee induced activation of these regions compared to non-painful stimulation. Additionally, we also observed differences in perception of electrical stimulation across four different body sites.

The repeated-measures ANOVA of the β values revealed that there was no significant main effect of stimulation across different body sites on oxyhemoglobin activity, aligning with our hypothesis. This result may be attributed to the adjacency of cortical projections for the bilateral groin and knees within S1, as well as the relatively small cortical representation of these lower limb regions^49^. Due to the spatial limitations of fNIRS, it is possible that the method was unable to detect significant differences in sensory cortical activation associated with stimulation of distinct lower limb regions. Additionally, we also found that the different levels of stimulation modality (painful and non-painful) did not significantly affect oxygenated hemodynamic activity, while the pattern of the oxygenated hemodynamic activity significantly depended on observed brain regions. From the supplement Table S7, it is evident that the β values of right S1 were higher than the β values of PFC. This phenomenon may be attributed to the specialization of the right hemisphere in the regulation of emotional and pain processing^50^, as well as the complexity of the PFC in interpreting sensory stimuli. These aspects will be clarified in the following discussion. Interestingly, the interaction between body sites, stimulation modalities, and brain regions significantly influenced oxygenated hemodynamic responses. This suggests that the combination of these factors exerts a complex modulatory effect on oxyhemoglobin activity, with the impact of different body sites potentially varying depending on the brain region and stimulation intensity. Consistent with the objectives of this study, the primary focus was on the differences in regional brain activation under painful and non-painful stimulation intensities. From the post-hoc tests, we observed that the PFC exhibited deactivation during painful stimulation compared to non-painful stimulation when electrical stimulation was applied to the left groin. This finding is in line with previous brain imaging studies. For instance, In an fMRI study, Kong et al.^51^ reported that high thermal pain stimulation applied to the right forearm of healthy persons induced bilateral deactivation of the medial prefrontal cortex. In an fNIRS study, Yücel et al.^21^ found that both acute electrical noxious and innocuous stimuli applied to the left thumb of healthy participants led to a significant decrease in HbO2 concentration in the superior and middle frontal gyrus. The deactivation was stronger in response to noxious stimuli, although this difference did not reach statistical significance. Similarly, our study observed deactivation in the PFC under painful stimulation, with significantly greater deactivation compared to non-painful stimulation. Notably, we also observed activation in the PFC under non-painful stimulation when the left groin was stimulated, this activation was not statistically significant. This observation may be related to the lack of subdivision of the PFC, a point we discuss in more detail later. Regarding the deactivation of this region, we can attribute it to the default mode network (DMN). The DMN comprises interactive brain areas that tend to show reduced activity when individuals are engaged in external tasks that require attention^52^. This network primarily involves the following three areas: ventromedial PFC (vmPFC), dorsomedial PFC (dmPFC), the posterior cingulate cortex (PCC), adjacent precuneus and angular gyrus^53^. One study demonstrated that pain-induced DMN deactivation is less pronounced when individuals shift their thoughts away from pain^54^. Another study exploring the analgesic effect of cold therapy noted that the group with negative experiences reported less pain under cold compression conditions compared to the group with positive experiences. This difference may be attributed to participants in the positive experience group directing more attention to their pain, resulting in the deactivation of the inferior parietal lobule^55^. Based on these observations, it is plausible to infer that as subjects receive different levels of electrical stimulation, higher stimulation intensities may lead to increased attention to pain, consequently resulting in more pronounced prefrontal cortex deactivation in the central region. Although no statistically significant differences were observed for the right groin and left knee, a consistent pattern is evident in Fig. 6 (b), which aligns with the significant findings from the left groin. Surprisingly, the results of stimulating the right knee in this study contradicted the hypothesis, revealing that prefrontal deactivation was more pronounced during non-painful stimulation. However, as shown in Fig. 6 (a), deactivation appeared to be focused more in the central part of the PFC, while activation appeared to be more lateralized in the PFC during painful stimulation compared to the non-stimulation periods at this site. This observation would suggest that a finer subdivision of the PFC cortical surface in more targeted regions of interest. In an fMRI study^56^, similar results were observed, indicating that individual differences in PFC activation during thermal stimulation of the lower leg in healthy individuals were localized near the lateral aspect of the superior frontal gyrus. We can attribute this result to the regulation of the salience network (SN). The SN is a large-scale network in the human brain involved in detecting and filtering significant stimuli, as well as engaging related functional networks. It integrates sensory, emotional, and cognitive information for various complex functions, including communication and self-awareness^57,58^. Consequently, when exposed to painful stimulation, the SN potentially establishes a functional pathway for interaction between the lateral PFC and the “somatosensory network”, enabling their participation in sensory detection, as well as higher-level integration and regulatory processes^59^. The outcomes of this study underscore the pivotal role of the prefrontal lobe in pain processing, suggesting that distinct regions within the prefrontal lobe exert varying regulatory influences on pain and sensory stimulation.

Interestingly, compared to non-painful stimulation, different lower limb regions revealed contrasting activation patterns in the bilateral S1 cortex under the painful stimulation. In detail, when right knee painful stimulation was administered, the neuro-metabolic activity of this brain region was significantly higher than that observed during non-painful stimulation. In contrast, the neuro-metabolic activity of the bilateral S1cortex was significantly lower during painful stimulation of the left groin compared to non-painful stimulation. The same activity pattern was observed in right S1 when painful stimulation was applied to the right groin. A prior meta-analytic study^2^ highlighted the activation of the sensory cortex in response to painful stimuli, employing various imaging modalities. Furthermore, it was observed that the S1 cortex predominantly processes signals of pain^10^. These could explain the observations when stimulating the right knee, but the results for the groin contradict these results. Although research on this specific site is limited, our further discussion of stimulation intensities across various body sites suggests that anatomical distinctions between the groin and knee may contribute to this outcome. Another study involving 15 healthy participants suggested that experimental groin pain may encompass the lower abdomen^60^. Consequently, we can hypothesize that the distribution and conduction of pain in response to painful stimuli differ between the groin and the knee, leading to reduced sensory cortex activation during painful stimulation. Additionally, it is important to note that the observed differences in activity patterns do not directly reflect the effect of stimulation at different body sites globally, instead they reflect the specific interaction effect. Therefore, further research is needed to elucidate the underlying mechanism.

In this study, it is evident that the stimulation intensity applied to the left groin was significantly higher than the one applied to the bilateral knees, indicating lower sensitivity to electrical stimulation at this body site. In the process of determining appropriate intensities for both painful and non-painful stimuli, we aimed to adjust stimulation intensities to elicit comparable perceived intensities across body sites. However, owing to the variability inherent in VAS ratings, our results revealed significant differences in the main effect across stimulated body sites when perception was assessed before and after the stimulation modality using the same VAS scale. Nevertheless, in the outcomes after multiple comparisons correction, no significant differences were observed among the four distinct body sites. Noteworthy, we observed a trend towards significance for higher ratings for the left knee in comparison to the right groin. One plausible explanation for these observations is that the knees exhibit a higher sensitivity compared to the groins. We assume that these differences may be attributed to variations in the related physiological and anatomical structures. The knee area contains a high concentration of nerve endings and a denser population of sensory neurons, rendering it more susceptible to irritation^61^. Additionally, nerves such as the femoral nerve, carry both sensory and motor signals and innervates the groin, front of the thigh, and knee regions^62^. Specifically, the anterior branch of the femoral nerve, responsible for sensory innervation, supplies the anterior medial skin regions of the distal thigh and the knee^62^, may play a role in this sensitivity. Therefore, we hypothesize that when stimulating the knee, due to the broader distribution of nerves in the knee area, including some passing through the groin region, we observe increased sensitivity in the knee. However, this increased sensitivity does not imply a difference in pain perception between the left and right knee. Additionally, in our study, we did not specifically hypothesize a lateral difference in sensitivity, and the absence of such a difference in reported stimulation perception may be attributed to the balanced experimental design, which randomized the order of stimulation.

This study has several limitations that should be acknowledged: (1) Limited age group: the participants recruited for this study were predominantly young individuals, averaging around 24 years of age. As a result, caution should be exercised when generalizing the findings of this study to different age groups. Additionally, we did not conduct a power calculation prior to recruitment. However, an a posteriori analysis indicated that our sample size of 16 participants provided approximately 80% power to detect large effect sizes (Cohen’s *d* = 0.8) and around 50% power to detect medium effect sizes (Cohen’s *d* = 0.5) at a significance level of 0.05. Future studies should conduct a power calculation prior to recruitment to ensure sufficient sample sizes. (2) Simplified analysis of fNIRS data: The analysis of our fNIRS data primarily relied on statistical analysis centered on beta values, focusing on spatial activation patterns. While this approach provides valuable insights, future research could benefit from incorporating time domain analysis techniques to delve deeper into cortical activation patterns and potential habituation effects when stimulating various lower limb regions. (3) Low spatial resolution: Given the spatial resolution limitations of fNIRS, the regional division method employed in this study and the use of average beta values may dilute localized effects and potentially reduce the overall effect. Future studies could address this issue by adopting finer regional divisions based on the framework established in this study or by focusing on representative individual channels for analysis. On the other hand, a channel-wise analysis of the hemodynamic responses would require additional measures to mitigate the risk of type I errors. (4) HbR concentration changes: The results obtained using the changes in concentration of HbR were not entirely consistent with those obtained with HbO2. The differences appear to rely on the biophysical modelling of the hemodynamic response of the two measures, which captures the two interconnected neuro-metabolic phenomena with different levels of sensitivity. Although an optimization of the HbR hemodynamic response would be required, this type of sensitivity analysis would entail additional investigations which are out of the scope of the present study. Nonetheless, this does not limit the validity of the statistical inference carried out using HbO2 data. (5) Exclusion of scalp hemodynamics: given the scarcity of prior studies focusing on lower limb regions and the limitations of fNIRS monitoring depth, this study aimed to capture hemodynamics across the entire cortex of interest. Therefore, we did not employ short separation channels to monitor scalp hemodynamics. In future experiments, incorporating this technique could help mitigate the potential impact of painful stimulation on hemodynamics in the target cortex. These limitations provide valuable insights into areas for potential refinement and expansion in future research endeavors.

In summary, our study demonstrates that stimulation at different lower limb regions does not significantly affect brain oxygen hemodynamic activity in healthy subjects subjected to acute electrical stimulation. However, the interaction of various factors, including stimulation site and intensity, plays a role in modulating brain activity, resulting in different activity patterns under specific stimulation modalities. This could be related to the brain activity of distinct regions within the PFC in response to painful stimulation, as well as the differential distribution and conduction of pain to the groin and knee. Additionally, our findings suggest that the knee exhibits greater sensitivity to sensory stimulation than the left groin. Consequently, our study contributes valuable evidence regarding the modulation of pain perception at the cortical level. Furthermore, it underscores the potential and feasibility of employing fNIRS to investigate pain mechanisms across diverse lower limb areas.

## Electronic supplementary material

Below is the link to the electronic supplementary material.

Supplementary Material 1

## Data Availability

The datasets can be obtained from the corresponding author upon reasonable request.

## References

[1] Espinoza, S. & Habas, C. Neuroimaging of pain. *Contemp. Clin. Neurosci.* **1**, 323–337. 10.1007/978-3-319-78926-2_14 (2018).

[2] Apkarian, A. V., Bushnell, M. C., Treede, R. D. & Zubieta, J. K. Human brain mechanisms of pain perception and regulation in health and disease. *Eur. J. Pain* **9**, 463 (2005). 15979027 10.1016/j.ejpain.2004.11.001

[3] Freund, W. et al. Perception and suppression of thermally induced pain: a fMRI study. *Somatosens. Mot. Res.* **26**, 1–10 (2009). 19283551 10.1080/08990220902738243

[4] Chen, T. L. et al. Human secondary somatosensory cortex is involved in the processing of somatosensory rare stimuli: an fMRI study. *Neuroimage* **40**, 1765–1771 (2008). 18329293 10.1016/j.neuroimage.2008.01.020

[5] Bornhövd, K. et al. Painful stimuli evoke different stimulus–response functions in the amygdala, prefrontal, insula and somatosensory cortex: a single-trial fMRI study. *Brain* **125**, 1326–1336 (2002). 12023321 10.1093/brain/awf137

[6] Sandström, A. et al. Altered cerebral pain processing of noxious stimuli from inflamed joints in rheumatoid arthritis: an event-related fMRI study. *Brain Behav. Immun.* **81**, 272–279 (2019). 31228612 10.1016/j.bbi.2019.06.024

[7] Tsuji, T. et al. Peripheral arterial stiffness during electrocutaneous stimulation is positively correlated with pain-related brain activity and subjective pain intensity: an fMRI study. *Sci. Rep.* **11**, 4425 (2021). 33627762 10.1038/s41598-021-83833-6 PMC7904817

[8] Ng, S. K. et al. The relationship between structural and functional brain changes and altered emotion and cognition in chronic low back pain brain changes: a systematic review of MRI and fMRI studies. *Clin. J. Pain* **34**, 237–261 (2018). 28719509 10.1097/AJP.0000000000000534

[9] Bushnell, M. C., Ceko, M. & Low, L. A. Cognitive and emotional control of pain and its disruption in chronic pain. *Nat. Rev. Neurosci.* **14**, 502–511 (2013). 23719569 10.1038/nrn3516 PMC4465351

[10] Vierck, C. J., Whitsel, B. L., Favorov, O. V., Brown, A. W. & Tommerdahl, M. Role of primary somatosensory cortex in the coding of pain. *Pain* **154**, 334–344 (2013). 23245864 10.1016/j.pain.2012.10.021 PMC4501501

[11] Kulkarni, B. et al. Attention to pain localization and unpleasantness discriminates the functions of the medial and lateral pain systems. *Eur. J. Neurosci.* **21**, 3133–3142 (2005). 15978022 10.1111/j.1460-9568.2005.04098.x

[12] Borsook, D. & Becerra, L. R. Breaking down the barriers: fMRI applications in pain, analgesia and analgesics. *Mol. Pain* **2**, 1 (2006). 16982005 10.1186/1744-8069-2-30 PMC1592305

[13] Glover, G. H. Overview of functional magnetic resonance imaging. *Neurosurg. Clin. N. Am.* **22**, 133–139 (2011). 21435566 10.1016/j.nec.2010.11.001 PMC3073717

[14] Chaudhary, U. et al. Motor response investigation in individuals with cerebral palsy using near infrared spectroscopy: pilot study. *Appl. Opt.* **53**, 503–510 (2014). 24514139 10.1364/AO.53.000503

[15] Ferrari, M. & Quaresima, V. A brief review on the history of human functional near-infrared spectroscopy (fNIRS) development and fields of application. *Neuroimage* **63**, 921–935 (2012). 22510258 10.1016/j.neuroimage.2012.03.049

[16] Chaudhary, U., Hall, M., DeCerce, J., Rey, G. & Godavarty, A. Frontal activation and connectivity using near-infrared spectroscopy: verbal fluency language study. *Brain Res. Bull.* **84**, 197–205 (2011). 21255633 10.1016/j.brainresbull.2011.01.002

[17] Eggebrecht, A. T. et al. Mapping distributed brain function and networks with diffuse optical tomography. *Nat. Photon.* **8**, 448–454 (2014). 10.1038/nphoton.2014.107 PMC4114252 25083161

[18] Becerra, L. et al. Diffuse optical tomography of pain and tactile stimulation: activation in cortical sensory and emotional systems. *Neuroimage* **41**, 252–259 (2008). 18394924 10.1016/j.neuroimage.2008.01.047 PMC2728450

[19] Lee, C. H. et al. Analysis for distinctive activation patterns of pain and itchy in the human brain cortex measured using near infrared spectroscopy (NIRS). *PLoS ONE* **8**, 1 (2013). 10.1371/journal.pone.0075360 PMC3789686 24098378

[20] Sakuma, S. et al. Experimental pain in the gingiva and its impact on prefrontal cortical hemodynamics: a functional near-infrared spectroscopy study. *Neurosci. Lett.* **575**, 74–79 (2014). 24878385 10.1016/j.neulet.2014.05.040

[21] Yücel, M. A. et al. Specificity of hemodynamic brain responses to painful stimuli: a functional near-infrared spectroscopy study. *Sci. Rep.* **5**, 1–9 (2015). 10.1038/srep09469 PMC4377554 25820289

[22] Aasted, C. M. et al. Frontal lobe hemodynamic responses to painful stimulation: a potential brain marker of nociception. *PLoS ONE* **11**, 1–12 (2016). 10.1371/journal.pone.0165226 PMC5091745 27806119

[23] Meyer-Frießem, C. H., Jess, G., Pogatzki-Zahn, E. M. & Zahn, P. K. Cerebral oxygenation for pain monitoring in adults is ineffective: a sequence-randomized, sham controlled study in volunteers. *Scand. J. Pain* **16**, 129–135 (2017). 28850388 10.1016/j.sjpain.2017.05.001

[24] Dreinhöfer, K. E., Reichel, H. & Käfer, W. Lower limb pain. *Best Pract. Res. Clin. Rheumatol.* **21**, 135–152 (2007). 17350549 10.1016/j.berh.2006.10.007

[25] Jørgensen, S. G., Öberg, S. & Rosenberg, J. Treatment of longstanding groin pain: a systematic review. *Hernia* **23**, 1035–1044 (2019). 30820781 10.1007/s10029-019-01919-7

[26] Voeller, G. Inguinal hernia management. *J. Am. Coll. Surg.* **197**, 702–703 (2003). 14522346 10.1016/S1072-7515(03)00604-5

[27] Bekrater-Bodmann, R., Reinhard, I., Diers, M., Fuchs, X. & Flor, H. Relationship of prosthesis ownership and phantom limb pain: results of a survey in 2383 limb amputees. *Pain* **162**, 630–640 (2021). 32868751 10.1097/j.pain.0000000000002063

[28] Kidd, V. D., Strum, S. R., Strum, D. S. & Shah, J. Genicular nerve radiofrequency ablation for painful knee arthritis: the why and the how. *JBJS Essent. Surg. Tech.* **9**, e10 (2019). 31333900 10.2106/JBJS.ST.18.00016 PMC6635137

[29] Öztürk, Ö., Algun, Z. C., Bombacı, H. & Erdoğan, S. B. Changes in prefrontal cortex activation with exercise in knee osteoarthritis patients with chronic pain: an fNIRS study. *J. Clin. Neurosci.* **90**, 144–151 (2021). 34275540 10.1016/j.jocn.2021.05.055

[30] Pollonini, L., Miao, H. & Ahn, H. Longitudinal effect of transcranial direct current stimulation on knee osteoarthritis patients measured by functional infrared spectroscopy: a pilot study. *Neurophotonics* **7**, 1 (2020). 10.1117/1.NPh.7.2.025004 PMC7203445 32411812

[31] Fuchs, X. et al. Phantom limb pain after unilateral arm amputation is associated with decreased heat pain thresholds in the face. *Eur. J. Pain (United Kingdom)* **26**, 114–132 (2022). 10.1002/ejp.1842 34288253

[32] Yennu, A., Tian, F., Gatchel, R. J. & Liu, H. Prefrontal hemodynamic mapping by functional near-infrared spectroscopy in response to thermal stimulations over three body sites. *Neurophotonics* **3**, 045008 (2016). 28018934 10.1117/1.NPh.3.4.045008 PMC5166717

[33] Hu, X. S., Nascimento, T. D. & DaSilva, A. F. Shedding light on pain for the clinic: a comprehensive review of using functional near-infrared spectroscopy to monitor its process in the brain. *Pain* **162**, 2805–2820 (2021). 33990114 10.1097/j.pain.0000000000002293 PMC8490487

[34] Williamson, A., Hoggart, B. & Pain A review of three commonly used pain rating scales. *J. Clin. Nurs.* **14**, 798–804 (2005). 16000093 10.1111/j.1365-2702.2005.01121.x

[35] Liu, H. et al. Peripheral input and phantom limb pain: a somatosensory event-related potential study. *Eur. J. Pain* **24**, 1314–1329 (2020). 32335979 10.1002/ejp.1579

[36] Löffler, M. et al. Impact of controllability on pain and suffering. *Pain Rep.* **3**, 1–10 (2018). 10.1097/PR9.0000000000000694 PMC6344140 30706037

[37] Zimeo Morais, G. A., Balardin, J. B. & Sato, J. R. FNIRS optodes’ location decider (fOLD): a toolbox for probe arrangement guided by brain regions-of-interest. *Sci. Rep.* **8**, 1–11 (2018). 29463928 10.1038/s41598-018-21716-z PMC5820343

[38] Rorden, C. & Brett, M. Stereotaxic display of brain lesions. *Behav. Neurol.* **12**, 191–200 (2000). 11568431 10.1155/2000/421719

[39] Piper, S. K. et al. A wearable multi-channel fNIRS system for brain imaging in freely moving subjects. *Neuroimage* **85**, 64–71 (2014). 23810973 10.1016/j.neuroimage.2013.06.062 PMC3859838

[40] Ye, J. C., Tak, S., Jang, K. E., Jung, J. & Jang, J. NIRS-SPM: statistical parametric mapping for near-infrared spectroscopy. *Neuroimage* **44**, 428–447 (2009). 18848897 10.1016/j.neuroimage.2008.08.036

[41] Shuvra, L. T., Rabiul Islam, M., Zaman, S. & Hasan, M. A. N. Identification of human pain perception using fNIRS. In *1st Int. Conf. Robot. Electr. Signal Process. Tech. ICREST 2019* 434–439. 10.1109/ICREST.2019.8644087 (2019).

[42] Friston, K. J. et al. Analysis of fMRI time-series revisited. *Neuroimage* **2**, 45–53 (1995). 9343589 10.1006/nimg.1995.1007

[43] Cohen, J. *Statistical Power Analysis for the Behavioral Sciences* 2nd edn. 10.4324/9780203771587 (Routledge, 1988).

[44] Wilcox, R. R. *Introduction to Robust Estimation and Hypothesis Testing* (Academic press, 2011).

[45] Xia, M., Wang, J. & He, Y. BrainNet viewer: a network visualization tool for human brain connectomics. *PLoS ONE* **8**, 1 (2013). 10.1371/journal.pone.0068910 PMC3701683 23861951

[46] Iso, N. et al. Hemodynamic signal changes during motor imagery task performance are associated with the degree of motor task learning. *Front. Hum. Neurosci.* **15**, 1–14 (2021). 10.3389/fnhum.2021.603069 PMC8081959 33935666

[47] Hoshi, Y., Kobayashi, N. & Tamura, M. Interpretation of near-infrared spectroscopy signals: a study with a newly developed perfused rat brain model. *J. Appl. Physiol.* **90**, 1657–1662 (2001). 11299252 10.1152/jappl.2001.90.5.1657

[48] Sun, Q. et al. Cortical activation patterns of different masking noises and correlation with their masking efficacy, determined by functional near-infrared spectroscopy. *Front. Hum. Neurosci.* **14**, 1–10 (2020). 32410973 10.3389/fnhum.2020.00149 PMC7198837

[49] Penpield, B. Y. W. somatic motor and sensory representation. In *Atlantic* (1937).

[50] Duerden, E. G. & Albanese, M. C. Localization of pain-related brain activation: a meta-analysis of neuroimaging data. *Hum. Brain Mapp.* **34**, 109–149 (2013). 22131304 10.1002/hbm.21416 PMC6869965

[51] Kong, J. et al. Exploring the brain in pain: activations, deactivations and their relation. *Pain* **148**, 257–267 (2010). 20005043 10.1016/j.pain.2009.11.008 PMC2815185

[52] Raichle, M. E. et al. A default mode of brain function. *Proc. Natl. Acad. Sci. U.S.A.* **98**, 676–682 (2001). 10.1073/pnas.98.2.676 PMC14647 11209064

[53] Raichle, M. E. The brain’s default Mode Network. *Annu. Rev. Neurosci.* **38**, 433–447 (2015). 25938726 10.1146/annurev-neuro-071013-014030

[54] Kucyi, A., Salomons, T. V. & Davis, K. D. Mind wandering away from pain dynamically engages antinociceptive and default mode brain networks. *Proc. Natl. Acad. Sci. U.S.A.* **110**, 18692–18697 (2013). 10.1073/pnas.1312902110 PMC3832014 24167282

[55] Choi, J. C. et al. The increased analgesic efficacy of cold therapy after an unsuccessful analgesic experience is associated with inferior parietal lobule activation. *Sci. Rep.* **12**, 14687 (2022). 36038625 10.1038/s41598-022-18181-0 PMC9424269

[56] Coghill, R. C., McHaffie, J. G. & Yen, Y. F. Neural correlates of interindividual differences in the subjective experience of pain. *Proc. Natl. Acad. Sci. U.S.A.* **100**, 8538–8542 (2003). 12824463 10.1073/pnas.1430684100 PMC166264

[57] Menon, V. & Uddin, L. Q. Saliency, switching, attention and control: a network model of insula function. *Brain Struct. Funct.* **214**, 655–667 (2010). 20512370 10.1007/s00429-010-0262-0 PMC2899886

[58] Peters, S. K., Dunlop, K. & Downar, J. Cortico-striatal-thalamic loop circuits of the salience network: a central pathway in psychiatric disease and treatment. *Front. Syst. Neurosci.* **10**, 104 (2016). 28082874 10.3389/fnsys.2016.00104 PMC5187454

[59] Peng, K., Steele, S. C., Becerra, L. & Borsook, D. Brodmann area 10: collating, integrating and high level processing of nociception and pain. *Prog. Neurobiol.* **161**, 1–22 (2018). 29199137 10.1016/j.pneurobio.2017.11.004 PMC5826795

[60] Drew, M. K. et al. Experimental pain in the groin may refer into the lower abdomen: implications to clinical assessments. *J. Sci. Med. Sport* **20**, 904–909 (2017). 28526226 10.1016/j.jsams.2017.04.007

[61] Horner, G. & Dellon, A. L. Innervation of the human knee joint and implications for surgery. *Clin. Orthop. Relat. Res.* **301**, 221–226 (1994). 8156678

[62] Kampitak, W. et al. The analgesic efficacy of anterior femoral cutaneous nerve block in combination with femoral triangle block in total knee arthroplasty: a randomized controlled trial. *Korean J. Anesthesiol.* **74**, 496–505 (2021). 34182749 10.4097/kja.21120 PMC8648511

