# Supplementary material for: Cortical activity during painful and non-painful stimulation over four lower limb body sites: a functional near-infrared spectroscopy study
Source: Sci Rep. 2025 Feb 11;15:5070. doi: 10.1038/s41598-025-87699-w (PMC11814190; doi:10.1038/s41598-025-87699-w)
Supplement: Supplementary file 1 — Supplementary Material 1 [file 41598_2025_87699_MOESM1_ESM.pdf]

Supplement

Cortical activity during painful and non-painful stimulation over four lower limb body sites: a functional near-infrared spectroscopy study

Jiawen Liao[1, *], Stefano Silvoni[1], Simon Desch[1, 2], Angela Serian[1], Jamila Andoh[1, 3], Herta Flor[1]

1. Department of Neuropsychology and Psychological Resilience Research, Central Institute of Mental Health, Medical Faculty Mannheim, Heidelberg University, Mannheim, Germany

2. Clinical Psychology, Department of Experimental Psychology, Heinrich Heine University Düsseldorf, Düsseldorf, Germany

3. Department of Psychiatry and Psychotherapy, Central Institute of Mental Health, Medical Faculty Mannheim, Heidelberg University, Mannheim, Germany

*Correspondence: Jiawen Liao, Department of Neuropsychology and Psychological Resilience Research, Central Institute of Mental Health, J 5, D-68159 Mannheim, Germany. E-mail: jiawen.liao@zi-mannheim.de

Table S1. Descriptive statistics and normality test results of stimulation intensities with a unit of milliamps (mA) over the two stimulation modalities and four different body sites.

Body sites	Stimulation modality	Number	Mean ± SD (mA)	Normality Test (*p*)
Left groin	Painful	16	1.62 ± 2.25	Not normal (*p* = 0.0004)
Left groin	Non-painful	16	0.68 ± 1.26	Not normal (*p* = 0.0000)
Right groin	Pain	16	0.81 ± 1.21	Not normal (*p* = 0.0002)
Right groin	Non-painful	16	0.22 ± 0.30	Not normal (*p* = 0.0002)
Left knee	Pain	16	0.20 ± 0.22	Not normal (*p* = 0.0011)
Left knee	Non-painful	16	0.07 ± 0.09	Not normal (*p* = 0.0002)
Right knee	Pain	16	0.26 ± 0.32	Not normal (*p* = 0.0003)
Right knee	Non-painful	16	0.12 ± 0.15	Not normal (*p* = 0.0001)

Note: Number represents sample sizes; SD means Standard deviation; Normality Test represents the result from Shapiro-Wilk normality test.

Table S2. Post-hoc multiple comparisons between the stimulation intensity of each body site.

(I)Body sites	(J)Body sites	Mean difference(I-J)	*p*	Cohen's *d*	95% Confidence Interval	
					Lower Bound	Upper Bound
Left groin	Right groin	0.63	0.0691	0.4326	-0.03	1.30
	Left knee	1.01	0.0007	0.7671	0.34	1.68
	Right knee	0.96	0.0015	0.7246	0.29	1.63
Right groin	Left groin	-0.63	0.0691	-0.4326	-1.30	0.03
	Left knee	0.38	0.4540	0.5734	-0.29	1.04
	Right knee	0.33	0.5789	0.4861	-0.34	0.99
Left knee	Left groin	-1.01	0.0007	-0.7671	-1.68	-0.34
	Right groin	-0.38	0.4540	-0.5734	-1.04	0.29
	Right knee	-0.05	0.9971	-0.2334	-0.72	0.62
Right knee	Left groin	-0.96	0.0015	-0.7246	-1.63	-0.29
	Right groin	-0.33	0.5789	-0.4861	-0.99	0.34
	Left knee	0.05	0.9971	0.2334	-0.62	0.72

Table S3. Descriptive statistics and normality test results of VAS ratings (ranging from "No sensation" to "Extreme pain," with values from 0 to 100) over the two stimulation modalities, two time points and four different body sites.

Body sites	Stimulation modality	Time	Number	Mean ± SD	Normality Test (p)
Left groin	Painful	Before	16	75.75 ± 7.30	Normal (p = 0.4660)
Left groin	Painful	After	16	75.88 ± 11.47	Not Normal (p = 0.0320)
Left groin	Non-painful	Before	16	23.88 ± 10.08	Normal (p = 0.6432)
Left groin	Non-painful	After	16	23.50 ± 14.30	Normal (p = 0.6355)
Right groin	Pain	Before	16	73.25 ± 8.06	Normal (p = 0.6013)
Right groin	Pain	After	16	76.25 ± 8.91	Normal (p = 0.4879)
Right groin	Non-painful	Before	16	22.00 ± 10.33	Normal (p = 0.1226)
Right groin	Non-painful	After	16	18.13 ± 11.72	Normal (p = 0.1031)
Left knee	Painful	Before	16	81.50 ± 6.35	Not Normal (p = 0.0060)
Left knee	Painful	After	16	84.13 ± 8.02	Normal (p = 0.6676)
Left knee	Non-painful	Before	16	27.00 ± 10.46	Normal (p = 0.6531)
Left knee	Non-painful	After	16	23.88 ± 14.56	Not Normal (p = 0.0332)
Right knee	Pain	Before	16	80.75 ± 6.57	Normal (p = 0.8161)
Right knee	Pain	After	16	79.38 ± 13.60	Not Normal (p = 0.0093)
Right knee	Non-painful	Before	16	25.63 ± 11.89	Normal (p = 0.5740)
Right knee	Non-painful	After	16	25.25 ± 13.80	Normal (p = 0.1927)

Note: Number represents sample sizes; SD means Standard deviation; Normality Test represents the result from Shapiro-Wilk normality test.

Table S4. Post-hoc multiple comparisons between the VAS ratings of each body site.

(I)Body sites	(J)Body sites	Mean difference(I-J)	p	Cohen's d	95% Confidence Interval	
					Lower Bound	Upper Bound
Left groin	Left knee	-4.38	0.2697	-1.8987	-11.02	2.27
	Right groin	2.34	0.4276	1.5627	-1.98	6.67
	Right knee	-3.00	0.6892	-1.1099	-10.79	4.79
Left knee	Left groin	4.38	0.2697	1.8987	-2.27	11.02
	Right groin	6.72	0.0507	2.8747	-0.02	13.45
	Right knee	1.38	0.8569	0.7921	-3.63	6.38
Right groin	Left groin	-2.34	0.4276	-1.5627	-6.67	1.98
	Left knee	-6.72	0.0507	-2.8747	-13.45	0.02
	Right knee	-5.34	0.2442	-1.9648	-13.18	2.50
Right knee	Left groin	3.00	0.6892	1.1099	-4.79	10.79
	Left knee	-1.38	0.8569	-0.7921	-6.38	3.63
	Right groin	5.34	0.2442	1.9648	-2.50	13.18

Table S5. Descriptive statistics and normality test results of β values (HbO2) over the two stimulation modalities, four different body sites and three brain regions.

Body sites	Stimulation modality	Brain region	Number	Mean ± SD ($\times 10^{-5}$)	Normality Test (*p*)
Left groin	Non-painful	Left S1	13	2.15 ± 0.81	Not normal (*p* = 0.0055)
Left groin	Non-painful	Right S1	13	3.10 ± 1.30	Not normal (*p* = 0.0021)
Left groin	Non-painful	PFC	13	0.85 ± 1.73	Not normal (*p* = 0.0031)
Left groin	Painful	Left S1	13	-2.20 ± 1.71	Not normal (*p* = 0.0345)
Left groin	Painful	Right S1	13	0.53 ± 2.50	Not normal (*p* = 0.0045)
Left groin	Painful	PFC	13	-3.91 ± 1.81	Normal (*p* = 0.3281)
Right groin	Non-painful	Left S1	16	2.05 ± 1.37	Normal (*p* = 0.8412)
Right groin	Non-painful	Right S1	16	4.70 ± 1.02	Normal (*p* = 0.0906)
Right groin	Non-painful	PFC	16	-0.32 ± 1.36	Normal (*p* = 0.8685)
Right groin	Painful	Left S1	16	2.51 ± 1.81	Not normal (*p* = 0.0328)
Right groin	Painful	Right S1	16	3.32 ± 1.15	Normal (*p* = 0.9613)
Right groin	Painful	PFC	16	-0.68 ± 1.65	Normal (*p* = 0.8918)
Left knee	Non-painful	Left S1	16	2.23 ± 1.25	Normal (*p* = 0.8633)
Left knee	Non-painful	Right S1	16	3.25 ± 2.97	Normal (*p* = 0.2854)
Left knee	Non-painful	PFC	16	-0.35 ± 1.49	Normal (*p* = 0.5882)
Left knee	Painful	Left S1	16	3.23 ± 1.49	Not normal (*p* = 0.0147)
Left knee	Painful	Right S1	16	3.80 ± 3.07	Not normal (*p* = 0.0145)
Left knee	Painful	PFC	16	-1.14 ± 2.95	Not normal (*p* = 0.0399)
Right knee	Non-painful	Left S1	15	-2.62 ± 1.88	Normal (*p* = 0.0787)
Right knee	Non-painful	Right S1	15	0.39 ± 1.57	Normal (*p* = 0.6343)
Right knee	Non-painful	PFC	15	-1.27 ± 2.36	Not normal (*p* = 0.0487)
Right knee	Painful	Left S1	15	2.92 ± 2.97	Normal (*p* = 0.0834)
Right knee	Painful	Right S1	15	4.79 ± 1.99	Normal (*p* = 0.4295)
Right knee	Painful	PFC	15	-0.33 ± 3.14	Normal (*p* = 0.3822)

Note: Number represents sample sizes; SD means Standard deviation; Normality Test represents the result from Shapiro-Wilk normality test.

Table S6. Table for β values (HbO2). Repeated-measures analysis of variance (ANOVA) with factors body sites, stimulation modality (non-painful, painful), brain groin (left S1, right S1 and PFC).

Source	Df	F	p	η^2
Body sites	3	0.16	0.9214	0.014
Stimulation modality	1	0.10	0.7609	0.009
Brain region	2	9.18	0.0013	0.455
Body sites*stimulation modality	3	1.23	0.3144	0.101
Body sites*brain region	6	0.69	0.6609	0.059
Stimulation modality*brain region	2	0.30	0.7460	0.026
Body sites*stimulation modality*brain region	6	3.12	0.0094	0.221

Note: Df means degree of freedom.

Table S7. Post-hoc multiple comparisons between the β values (HbO2) of each brain region.

(I)Brain region	(J)Brain region	Mean difference (I-J)	p	Cohen's d	95% confidence interval	
					Lower Bound	Upper Bound
Left S1	PFC	2.31E-05	0.0880	0.2177	-3.30E-06	4.96E-05
	Right S1	-2.09E-05	0.0851	-0.1662	-4.46E-05	2.78E-06
PFC	Left S1	-2.31E-05	0.0880	-0.2177	-4.96E-05	3.30E-06
	Right S1	-4.41E-05	0.0096	-0.4264	-7.66E-05	-1.16E-05
Right S1	Left S1	2.09E-05	0.0851	0.1662	-2.78E-06	4.46E-05
	PFC	4.41E-05	0.0096	0.4264	1.16E-05	7.66E-05

Table S8. Post-hoc multiple comparisons between the β values (HbO2) of different brain regions when stimulating different body sites.

Body sites	Brain regions	$Mean_{pain}$ ($\times 10^{-5}$)	$Mean_{non\text{-}pain}$ ($\times 10^{-5}$)	SD_{pain} ($\times 10^{-5}$)	$SD_{non\text{-}pain}$ ($\times 10^{-5}$)	p	Cohen's d
left groin	left S1	-2.20	2.15	1.71	0.81	**0.0002**	-3.2560
	right S1	0.53	3.10	2.50	1.30	**0.0096**	-1.2890
	PFC	-3.91	0.85	1.81	1.73	**<0.0001**	-2.6847
right groin	left S1	2.51	2.05	1.81	1.37	0.2746	0.2900
	right S1	3.32	4.70	1.15	1.02	**0.0185**	-1.2739
	PFC	-0.68	-0.32	1.65	1.36	0.1210	-0.2344
left knee	left S1	3.23	2.23	1.49	1.25	0.1559	0.7255
	right S1	3.80	3.25	3.07	2.97	0.4590	0.1816
	PFC	-1.14	-0.35	2.95	1.49	0.1537	-0.3372
right knee	left S1	2.92	-2.62	2.97	1.88	**0.0002**	2.2288
	right S1	4.79	0.39	1.99	1.57	**0.0002**	2.4612
	PFC	-0.33	-1.27	3.14	2.36	**0.0086**	0.3362

Note: SD means Standard deviation. **Bold *p*-values** indicate statistical significance after controlling for false discovery rate (FDR).

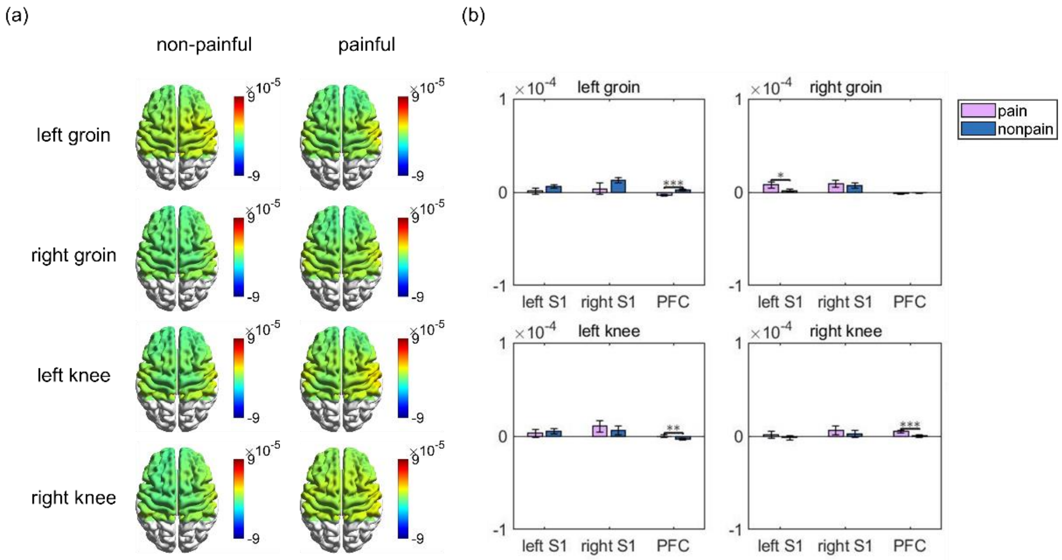

Figure S1. (a) Topographic images of group-averaged cortical deactivations and/or activations(HbR) during non-painful and painful stimulation modalities given on each body site. (b) Comparisons of the group-averaged β values (HbR) of different brain regions when stimulating different body sites. The pink bar represents the painful stimuli, and the blue bar represents the non-painful stimuli. Left S1 means left primary somatosensory area, right S1 means right primary somatosensory area, and PFC means prefrontal area. Error bars represent the standard error of the mean. (*= p<0.05, **= p<0.01 and ***= p<0.001).

Table S9. Comparisons of the group-averaged β values (HbR) of different brain regions when stimulating different body sites.

Body sites	Brain regions	$Mean_{pain}$ ($\times 10^{-5}$)	$Mean_{non\text{-}pain}$ ($\times 10^{-5}$)	SD_{pain} ($\times 10^{-5}$)	$SD_{non\text{-}pain}$ ($\times 10^{-5}$)	p	Cohen's d
left groin	left S1	0.11	0.66	0.90	0.50	0.0294	-0.7482
	right S1	0.38	1.26	1.61	0.77	0.0454	-0.6987
	PFC	-0.36	0.21	0.53	0.39	**<0.0001**	-1.2199
right groin	left S1	0.80	0.16	0.86	0.38	**0.0160**	0.9552
	right S1	0.90	0.68	0.91	0.79	0.3749	0.2596
	PFC	-0.16	-0.09	0.33	0.46	0.3795	-0.1753
left knee	left S1	0.31	0.53	1.08	0.76	0.3833	-0.2371
	right S1	1.04	0.62	1.62	1.28	0.1961	0.2909
	PFC	-0.03	-0.35	0.66	0.35	**0.0024**	0.5994
right knee	left S1	0.15	-0.15	0.95	0.61	0.2752	0.3750
	right S1	0.64	0.26	1.27	1.00	0.0892	0.3377
	PFC	0.49	0.02	0.71	0.66	**<0.0001**	0.6967

Note: SD means Standard deviation. **Bold *p*-values** indicate statistical significance after controlling for false discovery rate (FDR).

Table S10. Channel location and β values (HbO2) for per channel.

Channel	Source	Detector	X (mm)	Y (mm)	Z (mm)	Landmark	Left groin		Right groin		Left knee		Right knee	
							β (np)	β (p)	β (np)	β (p)	β (np)	β (p)	β (np)	β (p)
1	AF3	AFz	-12	62	23	10<br>9<br>46	-1.37E-05	-8.66E-05	-7.19E-06	-5.32E-06	-1.52E-05	-6.96E-06	-3.37E-05	-2.12E-06
2	AF3	F1	-23	52	32	9<br>46<br>10	-1.20E-05	-7.66E-05	-1.38E-05	-1.93E-05	-2.74E-05	-3.89E-05	-4.37E-05	-3.72E-05
3	AF3	F5	-39	50	17	46<br>45<br>10	1.34E-05	-4.21E-05	-9.08E-07	2.69E-06	-1.20E-05	1.98E-05	1.17E-06	2.90E-05
4	AF3	Fp1	-24	63	9	10<br>11<br>46	-1.10E-05	-6.44E-05	-2.06E-05	-1.98E-05	-2.52E-05	-6.22E-06	-2.07E-05	1.47E-05
5	AF4	AFz	13	61	24	10<br>9<br>46	-1.34E-05	-6.11E-05	-9.10E-06	5.64E-06	1.98E-06	-1.76E-06	-1.84E-05	9.42E-06
6	AF4	F2	22	52	33	9<br>46<br>10	-1.24E-05	-8.04E-05	-1.55E-05	-1.70E-05	-3.13E-06	-2.18E-05	-1.02E-05	-9.51E-06
7	AF4	F6	40	50	16	46<br>45<br>10	1.22E-05	-2.86E-05	-5.45E-07	4.59E-06	3.32E-05	3.53E-05	3.12E-05	4.37E-05
8	AF4	Fp2	25	63	9	10<br>11<br>46	-1.39E-05	-4.73E-05	-6.20E-06	-5.88E-06	1.03E-05	2.01E-05	1.80E-05	3.45E-05
9	C3	FC3	-50	-3	50	6<br>4<br>3<br>9	8.15E-06	-6.66E-05	1.99E-06	-5.95E-06	1.41E-05	8.28E-06	-4.40E-05	-2.40E-05
10	C3	C1	-42	-20	62	4<br>6<br>3	1.46E-05	-4.17E-05	5.18E-06	1.60E-05	2.87E-05	2.57E-05	-3.80E-05	6.19E-06
11	C3	C5	-60	-18	37	3<br>43<br>2<br>48<br>4<br>1	2.45E-05	-1.59E-05	2.15E-05	2.36E-05	3.83E-06	3.97E-05	-3.84E-05	4.01E-06
12	C3	CP3	-52	-34	52	40<br>2<br>3<br>1	3.29E-05	-2.24E-05	2.52E-05	1.74E-05	3.62E-05	3.90E-05	-1.60E-05	1.47E-05
13	C4	FC4	52	-4	48	6<br>4<br>3<br>9	2.56E-05	-3.65E-05	3.84E-05	1.24E-05	-1.15E-05	1.75E-07	7.82E-07	2.50E-05
14	C4	C2	42	-21	62	4<br>6<br>3	1.23E-05	-3.60E-05	4.06E-05	2.43E-05	2.45E-05	1.37E-05	-4.16E-06	9.01E-06
15	C4	C6	62	-20	37	2<br>3<br>43<br>1<br>48<br>4<br>40	1.76E-05	1.25E-05	3.92E-05	3.60E-05	-1.12E-06	2.73E-05	6.03E-06	3.94E-05
16	C4	CP4	53	-35	52	40<br>2<br>3<br>1	3.31E-05	-8.13E-06	6.72E-05	3.98E-05	5.56E-05	3.75E-05	1.62E-05	4.02E-05
17	Fz	AFz	2	50	39	9<br>10<br>8<br>32	9.72E-06	-4.26E-05	5.20E-06	-2.02E-06	3.40E-06	-2.14E-05	-2.13E-05	-3.05E-05
18	Fz	F1	-9	41	50	9<br>8	1.44E-05	-4.82E-05	-2.76E-06	7.51E-06	-8.97E-06	-5.37E-05	-3.30E-05	-6.87E-05
19	Fz	F2	10	41	50	9<br>8	1.47E-05	-4.91E-05	-4.90E-07	2.37E-06	-6.23E-06	-5.39E-05	-2.41E-05	-5.69E-05
20	Fz	FCz	1	27	58	8<br>6<br>9	3.15E-05	-3.51E-05	1.72E-05	2.65E-05	1.87E-07	-4.45E-05	-3.00E-05	-5.02E-05
21	F3	F1	-31	39	41	9<br>46	-1.67E-05	-5.77E-05	-1.30E-05	-2.80E-05	-7.61E-06	-3.92E-05	-3.73E-05	-5.03E-05
22	F3	F5	-46	39	26	45<br>46	3.19E-06	-5.01E-05	-5.21E-06	-1.68E-05	3.43E-06	-9.79E-06	-8.16E-06	-4.62E-06
23	F3	FC3	-45	25	41	9<br>44<br>45<br>46<br>6	-4.77E-07	-7.17E-05	-8.16E-06	-3.91E-05	-7.41E-06	-2.87E-05	-3.36E-05	-3.34E-05
24	F4	F2	30	40	41	9<br>46<br>8	9.20E-06	-6.04E-05	-5.99E-06	3.06E-06	-3.99E-06	-4.05E-05	-1.89E-05	-1.82E-05
25	F4	F6	46	38	24	45<br>46	1.54E-05	-4.06E-05	-3.32E-06	3.62E-06	8.05E-06	1.02E-05	1.06E-05	2.23E-05
26	F4	FC4	44	25	40	9<br>44<br>46<br>45<br>6	3.69E-05	-4.63E-05	2.13E-05	8.01E-07	1.90E-05	-1.53E-05	-4.24E-06	-1.10E-05
...														

Table S10. Channel location and β values (HbO2) for per channel (continued).

Channel	Source	Detector	X (mm)	Y (mm)	Z (mm)	Landmark	Left groin		Right groin		Left knee		Right knee	
							β (np)	β (p)	β (np)	β (p)	β (np)	β (p)	β (np)	β (p)
27	Fpz	AFz	1	64	14	10<br>9	-2.66E-05	-6.05E-05	-1.13E-05	-5.42E-06	-1.59E-05	-4.93E-06	-1.95E-05	-4.22E-06
28	Fpz	Fp1	-12	67	0	10<br>11	-3.74E-05	-6.51E-05	-2.54E-05	-3.47E-05	-2.90E-05	-1.40E-05	-3.47E-05	-1.85E-05
29	Fpz	Fp2	13	67	0	10<br>11	-2.31E-05	-5.82E-05	-3.59E-05	-2.40E-05	-3.37E-05	-3.06E-07	-1.42E-05	1.33E-05
30	AF7	F5	-47	46	6	45<br>46	2.26E-05	-2.51E-05	4.81E-06	-3.73E-06	4.52E-06	3.16E-05	1.27E-05	1.24E-05
31	AF7	Fp1	-33	59	-2	11<br>46<br>10<br>47	2.13E-06	-3.10E-05	-4.62E-07	-1.42E-06	-1.21E-05	1.90E-05	-1.07E-05	3.36E-06
32	AF8	F6	48	46	5	45<br>46<br>47	3.31E-05	9.52E-06	2.66E-05	2.92E-05	1.18E-05	4.76E-05	3.65E-05	4.96E-05
33	AF8	Fp2	34	59	-2	10<br>11<br>46<br>47	-1.61E-05	-4.14E-06	1.13E-05	7.04E-06	-4.03E-07	3.62E-05	3.42E-05	2.60E-05
34	CP1	C1	-27	-36	71	4<br>3<br>6<br>7<br>1<br>2<br>5	3.22E-05	7.59E-06	2.89E-05	4.30E-05	1.09E-05	2.54E-05	-2.17E-05	4.81E-05
35	CP1	CP3	-39	-48	60	40<br>7<br>2<br>3	2.89E-05	-1.17E-05	4.17E-05	4.48E-05	3.33E-05	3.16E-05	-1.72E-05	3.62E-05
36	CP2	C2	28	-36	71	4<br>3<br>6<br>7<br>1<br>2<br>5	3.76E-05	1.92E-05	4.35E-05	4.67E-05	3.89E-05	3.13E-05	-1.30E-05	2.44E-05
37	CP2	CP4	39	-49	60	40<br>7<br>2<br>3	3.80E-05	8.12E-06	4.92E-05	4.01E-05	5.85E-05	6.64E-05	2.47E-05	3.37E-05
38	CP5	C5	-63	-32	23	48<br>22<br>42<br>2<br>40	3.09E-05	-1.05E-05	1.89E-05	3.69E-05	2.89E-05	5.62E-05	1.16E-05	5.81E-05
39	CP5	CP3	-57	-48	38	40<br>39<br>48<br>22	3.75E-05	-1.45E-05	3.86E-05	1.64E-05	2.59E-05	4.41E-05	1.28E-05	3.91E-05
40	CP6	C6	65	-33	23	22<br>48<br>2<br>40<br>42	2.67E-05	1.81E-05	5.11E-05	3.32E-05	6.28E-05	8.97E-05	2.99E-05	8.22E-05
41	CP6	CP4	58	-48	38	40<br>39<br>48<br>22	4.88E-05	1.16E-05	4.89E-05	2.89E-05	6.96E-05	6.92E-05	4.19E-05	6.72E-05
42	FC2	F2	24	26	55	8<br>9<br>6	1.05E-05	-4.86E-05	7.15E-06	-8.17E-07	4.42E-07	-4.12E-05	-6.39E-06	-3.37E-05
43	FC2	FC4	39	12	54	6<br>9<br>8	1.42E-05	-4.04E-05	1.54E-05	-1.21E-05	1.03E-05	-2.18E-05	1.65E-05	-5.08E-06
44	FC2	C2	27	-4	68	6<br>8	7.85E-06	-3.65E-05	1.17E-05	6.64E-06	1.71E-05	-6.57E-07	-1.26E-05	-3.45E-06
45	FC2	FCZ	14	13	66	6<br>8	1.37E-05	-1.99E-05	1.91E-05	1.69E-05	1.49E-05	-1.56E-05	-1.38E-05	-2.55E-05
46	FC1	F1	-23	26	56	8<br>9<br>6	-6.37E-06	-6.75E-05	-8.07E-06	-2.17E-05	-1.18E-05	-5.52E-05	-3.86E-05	-5.69E-05
47	FC1	FC3	-38	12	55	6<br>9<br>8	-2.10E-05	-6.33E-05	-8.05E-06	-3.17E-05	1.09E-05	-3.07E-05	-1.81E-05	-2.78E-05
48	FC1	C1	-26	-5	68	6<br>8<br>4	2.89E-06	-5.22E-05	-5.10E-07	-1.38E-05	1.13E-05	-6.72E-06	-2.11E-05	5.10E-06
49	FC1	FCZ	-13	12	67	6<br>8	9.39E-06	-3.47E-05	1.86E-05	1.78E-05	-6.91E-07	-4.56E-05	-3.72E-05	-3.43E-05

. *Note*: X, Y, Z coordinates refer to the spatial locations in the brain based on the MNI coordinate system. Landmark represents the Brodmann area (1, 2, 3 - Primary Somatosensory Cortex; 4 - Primary Motor Cortex; 5, 7 - Somatosensory Association Cortex; 6 - Pre-Motor and Supplementary Motor Cortex; 8 - Includes Frontal eye fields; 9 - Dorsolateral prefrontal cortex; 10 - Frontopolar area; 11 - Orbitofrontal area; 22 - Superior Temporal Gyrus; 32 - Dorsal anterior cingulate cortex; 40 - Supramarginal gyrus part of Wernicke's area; 42 - Primary and Auditory Association Cortex; 43 - Subcentral area; 44 - pars opercularis, part of Broca's area; 45 - pars triangularis Broca's area; 46 - Dorsolateral prefrontal cortex; 47 - Inferior prefrontal gyrus; 48 - Retrosubicular area). β values (np) means the β values for the non-painful stimulation modality; β values (p) means the β values for the painful stimulation modality.
